# Electricity production and consumption data from Danish power grid and governmental office buildings

**DOI:** 10.1016/j.dib.2019.01.032

**Published:** 2019-01-19

**Authors:** Asger Alexander Wendt Karl, Esmir Maslesa, Morten Birkved

**Affiliations:** a Technical University of Denmark, Department of Civil Engineering, Biotechnology and Environmental Technology, Denmark; b Technical University of Denmark, Department of Management Engineering, Biotechnology and Environmental Technology, Denmark; c University of Southern Denmark, SDU Life Cycle Engineering, Department of Chemical Engineering, Biotechnology and Environmental Technology, Denmark

## Abstract

This data article is the second component of the two-part study on the effects of high-resolution energy data on building life cycle assessments (LCAs) (Environmental performance assessment of the use stage of buildings using dynamic high-resolution energy consumption and data on grid composition, in press) [1]. The first part being the article serving as the main platform for the communication of the conclusions of the study, and this data article serving as Supporting information, as well as a means to present any results and analysis not included in the article. The focus is to present additional information strengthening the findings of the article, by showing data, graphs, and tables not included in the research article due to size limitations.

**Specifications table**

**Table:** 

Subject area	*Building, Energy*
More specific subject area	*Electricity consumption, Use stage*
Type of data	*Table and figure*
How data was acquired	*National energy grid database, special agreement with governmental agency BYGST*
Data format	*Raw*
Experimental factors	*Data selected based on availability of hourly resolution, and representation of buildings of varying size and age.*
Experimental features	*LCA analysis*
Data source location	*Nationwide, Denmark, building locations anonymous.*
Data accessibility	*Partly public, partly confidential. Results published are publicly available, parts of data used are confidential I.E. data concerning the buildings.*
Related research article	*“Environmental performance assessment of the use stage of buildings using dynamic high-resolution energy consumption and data on grid composition” – in press*

**Value of the data**

• Documentation of substantial variance in electrical grid and associated impact potentials
• Comparison between various data-resolutions shows deviation in environmental impacts
• Highlights shortcomings of conventional, static system models

## 1 Data

– *National grid composition data (Denmark, 2017)*
– *Electricity consumption data (18 office buildings in DK, 2017)*
– *Reference database (ecoinvent 3.3)*

Presented in this article are figures and tables showing the most important results of the data analysis, both from analysis of the separate data sets as well as the results from combining the two.

**Fig. 1:**
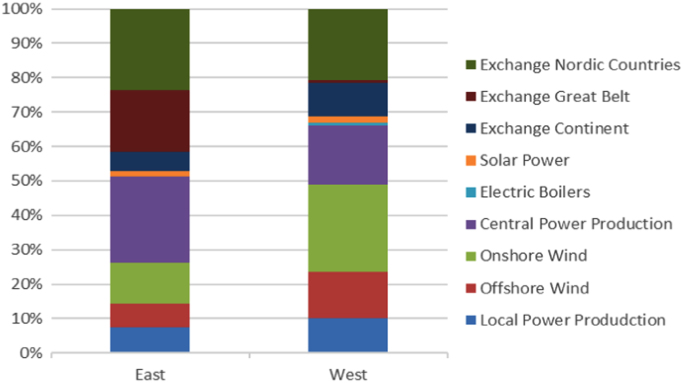
Average yearly electricity grid compositions, for east and west Denmark.

**Fig. 2:**
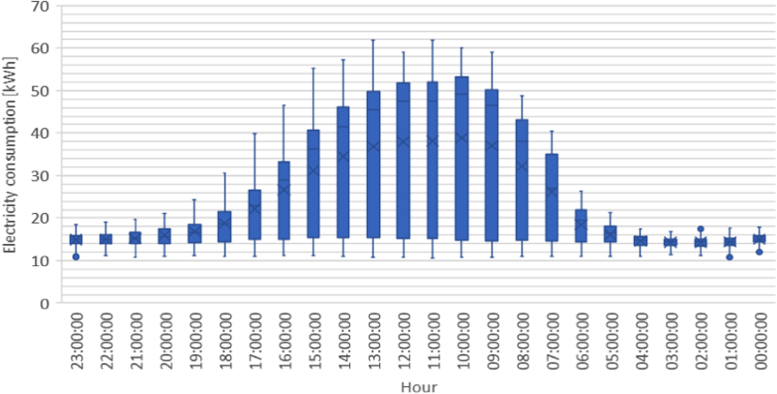
Average daily electricity consumption throughout the week, east Denmark in blue and west Denmark in red.

**Fig. 3:**
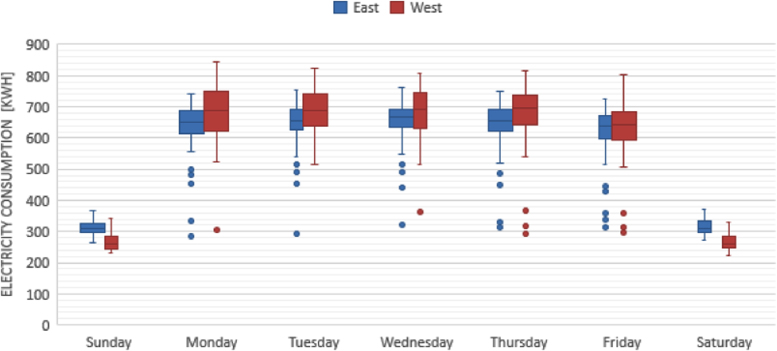
Average hourly distribution of electricity consumption.

**Fig. 4:**
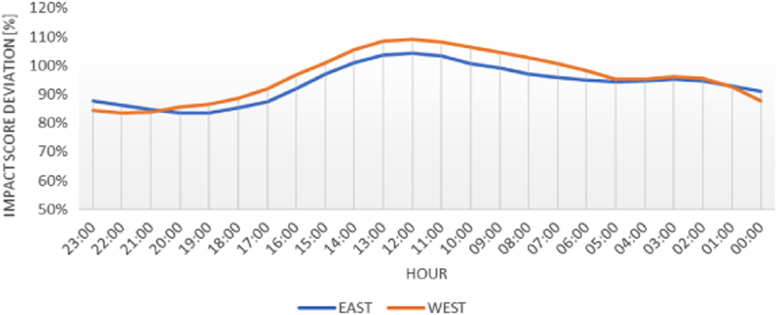
Average hourly deviation in impact potentials pr. kWh.

The figures presented in the following section describe the initial findings of the analysis, and show the spatial and temporal variations found within each of the data sets. Fig. 1, Fig. 4 display the results from the electricity production data set, and show the composition of the electrical grid as it varies both in location and over time. Fig. 2, Fig. 3 present the average temporal variations found in the building consumption data, as it changes throughout a day and a week.

The tables show the progress of the analysis as it was carried out, with the initial table displaying the source specific reference impact potentials, which lay the foundation on which the actual impact potentials are calculated and presented in the following tables. These tables show the monthly average deviations in impact potentials pr. kWh, once for western Denmark and once for eastern Denmark. Lastly the actual environmental impact scores are presented, calculated from the combination of the electrical grid data with the actual building consumption data. The scores are presented as impact potentials distributed among the age groupings of the buildings in the study, once for each data resolution used (as well as the reference process calculations) and lastly a comparison between each resolution system, showing the variation in results based on data complexity.

## 2 Experimental design, materials, and methods

The electrical grid composition data are publicly available, showing the sources comprising the electricity production for every hour, in east and west Denmark [2]. The data concerning the electricity consumption of the selected buildings are however not publicly available, and were only usable after a special agreement with Bygningsstyrelsen (The Danish Building and Property Agency), and are as such not included in this data article.

With the composition of the electrical grid known for every hour of 2017, the environmental impact potentials of one kWh for each energy source was determined using openLCA and the ecoinvent 3.3 database. These impact potentials are displayed in Table 1.

**Table 1:** Reference source specific unit impacts potentials from openLCA, showing the impacts pr. kWh for every electricity production source.

From the electrical grid composition data, the differences between east and west were determined, as well as the variations in the composition by hour, day, and month. These differences are displayed in Fig. 1, Fig. 2, Fig. 3 (Table 2, Table 3, Table 4, Table 5, Table 6, Table 7, Table 8).

**Table 2:** Impact potentials pr. kWh by months relative to the yearly average, east Denmark - Hourly. The months with impacts significantly lower than the yearly average are denoted by a green color, while the higher scores are presented as orange/red.

**Table 3:** Impact potentials pr. kWh by months relative to the yearly average. west Denmark - Hourly. The months with impacts significantly lower than the yearly average are denoted by a green color. while the higher scores are presented as orange/red.

**Table 4:** Midpoint impact score results from analysis of hourly grid resolution.

*Hourly grid resolution*	*W-II*	*W-II*	*W-III*	*E-I*	*E-II*	*EI-III*	*Unit*
*Fossil depletion*	2.64E + 00	2.59E + 00	1.85E + 00	2.00E + 00	1.69E + 00	1.56E + 00	kg oil eq /m2
*Marine eutrophication*	2.49E−03	2.45E − 03	1.75E − 03	1.80E − 03	1.52E − 03	1.40E − 03	kg N eq /m2
*Terrestrial acidification*	4.00E − 02	3.86E-02	2.78E − 02	3.28E − 02	2.78E − 02	2.57E − 02	kg SO2 eq /m2
*Ionizing radiation*	1.04E + 00	1.04E + 00	7.34E − 01	6.58E − 01	5.53E − 01	5.02E − 01	kg U235 eq /m2
*Freshwater ecotoxicity*	1.71E − 01	1.68E − 01	1.19E − 01	1.02E − 01	8.64E − 02	7.86E − 02	kg 1.4-DB eq /m2
*Photochemical oxidant formation*	2.71E − 02	2.63E − 02	1.89E − 02	2.06E − 02	1.75E − 02	1.61E − 02	kg NMVOC /m2
*Terrestrial ecotoxicity*	1.45E − 03	1.39E − 03	9.91E − 04	1.03E − 03	8.75E − 04	8.01E − 04	kg 1.4-DB eq /m2
*Marine ecotoxicity*	1.61E − 01	1.58E − 01	1.12E − 01	9.75E − 02	8.22E − 02	7.48E − 02	kg 1.4-DB eq /m2
*Climate Change*	1.01E + 01	9.84E + 00	7.04E + 00	7.95E + 00	6.75E + 00	6.21E + 00	kg CO2 eq /m2
*Particulate matter formation*	1.31E − 02	1.26E − 02	9.06E − 03	1.05E − 02	8.93E − 03	8.22E − 03	kg PM10 eq /m2
*Human toxicity*	4.45E + 00	4.41E + 00	3.13E + 00	3.10E + 00	2.62E + 00	2.40E + 00	kg 1.4-DB eq /m2
*Metal depletion*	3.78E − 01	3.69E − 01	2.61E − 01	2.29E − 01	1.93E − 01	1.76E − 01	kg Fe eq /m2
*Natural land transformation*	1.43E − 03	1.39E − 03	9.97E − 04	1.12E − 03	9.45E − 04	8.66E − 04	m2 /m2
*Urban land occupation*	2.15E − 01	2.07E − 01	1.49E − 01	1.85E − 01	1.57E − 01	1.45E − 01	m2*a /m2
*Water depletion*	2.90E + 01	2.87E + 01	2.01E + 01	2.93E + 01	2.46E + 01	2.21E + 01	m3 / m2
*Freshwater eutrophication*	5.89E − 03	5.89E − 03	4.17E − 03	3.89E − 03	3.29E − 03	3.01E − 03	kg P eq /m2
*Ozone depletion*	1.35E − 06	1.31E − 06	9.39E − 07	1.01E − 06	8.61E − 07	7.93E − 07	kg CFC-11 eq /m2
*Agricultural land occupation*	1.63E + 01	1.57E + 01	1.13E + 01	1.44E + 01	1.23E + 01	1.14E + 01	m2*a /m2

**Table 5:** Midpoint impact score results from analysis of daily grid resolution.

*Daily grid resolution*	*WI*	*WII*	*WIII*	*EI*	*EII*	*EIII*	*Unit*
*Fossil depletion*	2.67E + 00	2.56E + 00	1.87E + 00	2.01E + 00	1.70E + 00	1.52E + 00	kg oil eq /m2
*Marine eutrophication*	2.51E − 03	2.42E − 03	1.77E − 03	1.80E − 03	1.53E − 03	1.36E − 03	kg N eq /m2
*Terrestrial acidification*	4.01E − 02	3.81E − 02	2.80E − 02	3.27E − 02	2.79E − 02	2.48E − 02	kg SO2 eq /m2
*Ionizing radiation*	1.06E + 00	1.03E + 00	7.48E − 01	6.70E − 01	5.64E − 01	5.04E − 01	kg U235 eq /m2
*Freshwater ecotoxicity*	1.70E − 01	1.65E − 01	1.19E − 01	1.02E − 01	8.63E − 02	7.73E − 02	kg 1.4-DB eq /m2
*Photochemical oxidant formation*	2.73E − 02	2.59E − 02	1.90E − 02	2.21E − 02	1.88E − 02	1.67E − 02	kg NMVOC /m2
*Terrestrial ecotoxicity*	1.39E − 03	1.33E − 03	9.65E − 04	1.01E − 03	8.54E − 04	7.60E − 04	kg 1.4-DB eq /m2
*Marine ecotoxicity*	1.60E − 01	1.55E − 01	1.12E − 01	9.73E − 02	8.21E − 02	7.35E − 02	kg 1.4-DB eq /m2
*Climate Change*	1.02E + 01	9.76E + 00	7.15E + 00	7.96E + 00	6.77E + 00	6.03E + 00	kg CO2 eq /m2
*Particulate matter formation*	1.23E − 02	1.17E − 02	8.58E − 03	1.01E − 02	8.59E − 03	7.65E − 03	kg PM10 eq /m2
*Human toxicity*	4.49E + 00	4.37E + 00	3.19E + 00	3.10E + 00	2.62E + 00	2.34E + 00	kg 1.4-DB eq /m2
*Metal depletion*	3.77E − 01	3.62E − 01	2.62E − 01	2.34E − 01	1.96E − 01	1.76E − 01	kg Fe eq /m2
*Natural land transformation*	1.44E − 03	1.37E − 03	1.00E − 03	1.13E − 03	9.55E − 04	8.50E − 04	m2 /m2
*Urban land occupation*	2.17E − 01	2.05E − 01	1.51E − 01	1.84E − 01	1.58E − 01	1.40E − 01	m2*a /m2
*Water depletion*	3.05E + 01	2.94E + 01	2.12E + 01	2.94E + 01	2.47E + 01	2.20E + 01	m3 / m2
*Freshwater eutrophication*	5.99E − 03	5.87E − 03	4.28E − 03	3.91E − 03	3.31E − 03	2.96E − 03	kg P eq /m2
*Ozone depletion*	1.35E − 06	1.28E − 06	9.38E − 07	1.02E − 06	8.65E − 07	7.70E − 07	kg CFC-11 eq /m2
*Agricultural land occupation*	1.64E + 01	1.55E + 01	1.14E + 01	1.44E + 01	1.23E + 01	1.09E + 01	m2*a /m2

**Table 6:** Midpoint impact score results from analysis of monthly grid resolution.

*Monthly grid resolution*	*WI*	*WII*	*WIII*	*EI*	*EII*	*EIII*	*Unit*
*Fossil depletion*	2.76E + 00	2.62E + 00	1.91E + 00	2.04E + 00	1.73E + 00	1.55E + 00	kg oil eq /m2
*Marine eutrophication*	2.66E − 03	2.52E − 03	1.84E − 03	1.86E − 03	1.58E − 03	1.40E − 03	kg N eq /m2
*Terrestrial acidification*	3.97E − 02	3.77E − 02	2.76E − 02	3.25E − 02	2.77E − 02	2.47E − 02	kg SO2 eq /m2
*Ionizing radiation*	1.17E + 00	1.11E + 00	8.08E − 01	7.17E − 01	6.01E − 01	5.37E − 01	kg U235 eq /m2
*Freshwater ecotoxicity*	1.84E − 01	1.74E − 01	1.27E − 01	1.07E − 01	9.03E − 02	8.06E − 02	kg 1.4-DB eq /m2
*Photochemical oxidant formation*	2.70E − 02	2.57E − 02	1.88E − 02	2.20E − 02	1.87E − 02	1.67E − 02	kg NMVOC /m2
*Terrestrial ecotoxicity*	1.39E − 03	1.33E − 03	9.66E − 04	1.00E − 03	8.50E − 04	7.57E − 04	kg 1.4-DB eq /m2
*Marine ecotoxicity*	1.72E − 01	1.64E − 01	1.19E − 01	1.02E − 01	8.58E − 02	7.66E − 02	kg 1.4-DB eq /m2
*Climate Change*	1.05E + 01	9.94E + 00	7.27E + 00	8.08E + 00	6.88E + 00	6.13E + 00	kg CO2 eq /m2
*Particulate matter formation*	1.22E − 02	1.16E − 02	8.49E − 03	1.01E − 02	8.57E − 03	7.63E − 03	kg PM10 eq /m2
*Human toxicity*	4.89E + 00	4.63E + 00	3.38E + 00	3.25E + 00	2.75E + 00	2.46E + 00	kg 1.4-DB eq /m2
*Metal depletion*	3.92E − 01	3.73E − 01	2.71E − 01	2.37E − 01	1.99E − 01	1.78E − 01	kg Fe eq /m2
*Natural land transformation*	1.45E − 03	1.38E − 03	1.00E − 03	1.13E − 03	9.57E − 04	8.52E − 04	m2 /m2
*Urban land occupation*	2.11E − 01	2.00E − 01	1.47E − 01	1.82E − 01	1.56E − 01	1.38E − 01	m2*a /m2
*Water depletion*	3.13E + 01	2.99E + 01	2.15E + 01	3.01E + 01	2.53E + 01	2.25E + 01	m3 / m2
*Freshwater eutrophication*	6.70E − 03	6.35E − 03	4.64E − 03	4.21E − 03	3.55E − 03	3.17E − 03	kg P eq /m2
*Ozone depletion*	1.35E − 06	1.28E − 06	9.39E − 07	1.02E − 06	8.65E − 07	7.70E − 07	kg CFC-11 eq /m2
*Agricultural land occupation*	1.58E + 01	1.50E + 01	1.10E + 01	1.41E + 01	1.21E + 01	1.07E + 01	m2*a /m2

**Table 7:** Midpoint impact score results from analysis of reference grid.

*Reference grid*	*WI*	*WII*	*WIII*	*EI*	*EII*	*EIII*	*Unit*
*Fossil depletion*	4.80E + 00	4.57E + 00	3.32E + 00	3.76E + 00	3.17E + 00	2.83E + 00	kg oil eq /m2
*Marine eutrophication*	3.49E − 03	3.32E − 03	2.41E − 03	2.73E − 03	2.31E − 03	2.06E − 03	kg N eq /m2
*Terrestrial acidification*	6.05E − 02	5.76E − 02	4.18E − 02	4.74E − 02	4.00E − 02	3.56E − 02	kg SO2 eq /m2
*Ionizing radiation*	5.82E + 00	5.54E + 00	4.02E + 00	4.56E + 00	3.85E + 00	3.43E + 00	kg U235 eq /m2
*Freshwater ecotoxicity*	1.03E + 00	9.78E − 01	7.10E − 01	8.06E − 01	6.80E − 01	6.06E − 01	kg 1.4-DB eq /m2
*Photochemical oxidant formation*	3.50E − 02	3.33E − 02	2.42E − 02	2.74E − 02	2.31E − 02	2.06E − 02	kg NMVOC /m2
*Terrestrial ecotoxicity*	2.77E − 03	2.63E − 03	1.91E − 03	2.17E − 03	1.83E − 03	1.63E − 03	kg 1.4-DB eq /m2
*Marine ecotoxicity*	9.07E − 01	8.63E − 01	6.27E − 01	7.11E − 01	6.00E − 01	5.35E − 01	kg 1.4-DB eq /m2
*Climate Change*	1.89E + 01	1.80E + 01	1.31E + 01	1.48E + 01	1.25E + 01	1.11E + 01	kg CO2 eq /m2
*Particulate matter formation*	1.90E − 02	1.81E − 02	1.31E − 02	1.49E − 02	1.26E − 02	1.12E − 02	kg PM10 eq /m2
*Human toxicity*	7.20E + 00	6.85E + 00	4.97E + 00	5.64E + 00	4.76E + 00	4.24E + 00	kg 1.4-DB eq /m2
*Metal depletion*	8.57E − 01	8.16E − 01	5.92E − 01	6.72E − 01	5.67E − 01	5.05E − 01	kg Fe eq /m2
*Natural land transformation*	2.64E − 03	2.51E − 03	1.82E − 03	2.07E − 03	1.74E − 03	1.55E − 03	m2 /m2
*Urban land occupation*	2.38E − 01	2.26E − 01	1.64E − 01	1.87E − 01	1.57E − 01	1.40E − 01	m2*a /m2
*Water depletion*	2.10E + 02	2.00E + 02	1.45E + 02	1.64E + 02	1.39E + 02	1.24E + 02	m3 / m2
*Freshwater eutrophication*	8.00E − 03	7.61E − 03	5.53E − 03	6.27E − 03	5.29E − 03	4.71E − 03	kg P eq /m2
*Ozone depletion*	2.42E − 06	2.31E − 06	1.67E − 06	1.90E − 06	1.60E − 06	1.43E − 06	kg CFC-11 eq /m2
*Agricultural land occupation*	1.15E + 01	1.10E + 01	7.96E + 00	9.03E + 00	7.62E + 00	6.79E + 00	m2*a /m2

**Table 8:** Average yearly midpoint scores pr. kWh for western Denmark, calculated from the hourly grid resolution. And compared to the scores from the other resolutions, as well as the reference grid.

*Resolution comparisons*	*unit*	*Hourly*	*Daily*	*Monthly*	*Yearly*	*Reference*
*Fossil depletion*	kg oil eq /kWh	5.40E − 02	100.03%	99.03%	108.99%	177.80%
*Marine eutrophication*	kg N eq /kWh	5.20E − 05	100.17%	99.47%	116.69%	134.07%
*Terrestrial acidification*	kg SO2 eq /kWh	7.76E − 04	99.91%	98.52%	91.31%	155.96%
*Ionizing radiation*	kg U235 eq /kWh	2.31E − 02	100.15%	99.60%	134.54%	503.59%
*Freshwater ecotoxicity*	kg 1.4-DB eq /kWh	3.61E − 03	100.68%	101.37%	115.85%	569.38%
*Photochemical oxidant formation*	kg NMVOC /kWh	5.29E − 04	99.91%	98.58%	92.49%	132.37%
*Terrestrial ecotoxicity*	kg 1.4-DB eq /kWh	2.74E − 05	100.39%	99.01%	84.71%	201.89%
*Marine ecotoxicity*	kg 1.4-DB eq /kWh	3.39E − 03	100.66%	101.26%	116.09%	535.26%
*Climate Change*	kg CO2 eq /kWh	2.05E − 01	100.07%	99.04%	109.37%	184.64%
*Particulate matter formation*	kg PM10 eq /kWh	2.56E − 04	93.35%	92.00%	94.05%	148.66%
*Human toxicity*	kg 1.4-DB eq /kWh	9.57E − 02	100.41%	100.12%	127.74%	150.48%
*Metal depletion*	kg Fe eq /kWh	7.76E − 03	100.35%	101.31%	98.29%	220.92%
*Natural land transformation*	m2 /kWh	2.86E − 05	99.76%	98.25%	97.71%	184.48%
*Urban land occupation*	m2*a /kWh	4.11E − 03	99.89%	98.55%	85.90%	115.75%
*Water depletion*	m3 /kWh	6.28E − 01	100.26%	98.47%	134.16%	668.88%
*Freshwater eutrophication*	kg P eq /kWh	1.31E − 04	100.41%	100.22%	138.93%	121.90%
*Ozone depletion*	kg CFC-11 eq /kWh	2.65E − 08	99.83%	98.46%	93.07%	182.88%
*Agricultural land occupation*	m2*a /kWh	3.07E − 01	99.75%	97.93%	80.96%	75.06%

With the grid compositions and source specific impact potentials known, the actual environmental impact pr. kWh for any given hour, day, and month can be determined. These results are later combined with the electricity consumption for the selected buildings, to calculate the environmental performance as accurately as possible.
