# Supplementary material for: Electricity production and consumption data from Danish power grid and governmental office buildings
Source: Data Brief. 2019 Jan 19;23:103684. doi: 10.1016/j.dib.2019.01.032 (PMC6369333; doi:10.1016/j.dib.2019.01.032)
Supplement: Supplementary file 2 — Supplementary material [file mmc2.docx]

**Supporting information**

Asger Alexander Wendt Karl^*,1^, Esmir Maslesa^**^, Morten Birkved^**^

^*^ Technical University of Denmark, Department of Civil Engineering, Lyngby, Denmark.

^**^ Technical University of Denmark, Department of Management Engineering, Lyngby, Denmark.

^1)^ Email: AsgerWKarl@gmail.com

Table of contents

[3 Introduction 47](#_Toc515627151)

[4 Results 48](#_Toc515627152)

[4.1 Electric grid results 48](#_Toc515627153)

[4.2 Building electricity results 54](#_Toc515627154)

[4.3 Electrical grid compositions 56](#_Toc515627155)

[4.3.1 Dynamic high-resolution grid 56](#_Toc515627156)

[4.3.2 Static reference grid 57](#_Toc515627157)

[5 Analysis 58](#_Toc515627158)

[5.1 Hourly grid resolution 58](#_Toc515627159)

[5.2 Daily grid resolution 60](#_Toc515627160)

[5.3 Monthly grid resolution 61](#_Toc515627161)

[5.4 Comparison 62](#_Toc515627162)

[5.4.1 Midpoint results 62](#_Toc515627163)

[5.4.2 Endpoint results 64](#_Toc515627164)

[5.4.3 Environmental performances 65](#_Toc515627165)

[6 Appendix A – midpoint scores 70](#_Toc515627166)

[6.1 Hourly grid resolution 70](#_Toc515627167)

[6.2 Daily grid resolution 71](#_Toc515627168)

[6.3 Monthly grid resolution 72](#_Toc515627169)

[6.4 Reference grid 73](#_Toc515627170)

[7 Appendix B – Endpoint scores 74](#_Toc515627171)

[7.1 Hourly grid resolution 74](#_Toc515627172)

[7.2 Daily grid resolution 74](#_Toc515627173)

[7.3 Monthly grid resolution 74](#_Toc515627174)

[7.4 Reference grid 74](#_Toc515627175)

# 1 Introduction

This data article is the second component of the two-part study on the effects of high-resolution energy data on building life cycle assessments (LCAs). The first part being the article serving as the main platform for the communication of the conclusions of the study, and this data article serving as supporting information as well as a means to present any results and analysis not included in the article.

The focus of the data article is twofold; firstly to present additional information strengthening the findings of the article, by showing data, graphs, and tables not included in the article due to size limitations. And secondly to present conclusions that pertain to the variations in the buildings, and shifts the focus more from the electrical grid data analysis to the environmental building performances (EBP) as it shifts with building size and age.

The overarching purpose of the study is to examine the effects of utilizing dynamic high-resolution electricity data, matched with building electricity consumption data of equal resolutions. The study incorporates dynamic data showing variations in the electrical grid over time, as it changes by the hour throughout the year 2017 in Denmark, split into eastern and western sections. This electrical grid data is then coupled with data concerning the electricity consumption of building supplied by the grid, also with a resolution of one datapoint pr. hour. The buildings chosen for the study are equally distributed in east and west, with nine buildings in each section for a total of 18, within each sector (east/west) the buildings are selected to represent differences in age and area, in order to determine if these factors change the nature of the findings.

The aim of the study is to determine how representative current building LCA practices are, and if high-resolution data coupled with dynamic modelling practices provides results that differ in a meaningful way compared to current standard practices. This aim can be represented as a number of research questions:

1. How does the electrical grid, and the impacts associated with it, change throughout the year, and to what degree?
2. Are there major differences between eastern and western Denmark?
3. How does the electrical consumption of buildings vary over the course of a year?
4. What is the impact of age and/or area on the energy performance of a building?
5. How do the impact score results differ between various dynamic data resolutions?
6. What is the difference in results from a dynamic high-resolution system to the standard reference system?

These are factors not included in the static modelling process of EBP, it is through the answering of these questions that the study aims to assess the potential influence of these factors on building LCAs as a whole.

# 2 Results

## 2.1 Electric grid results


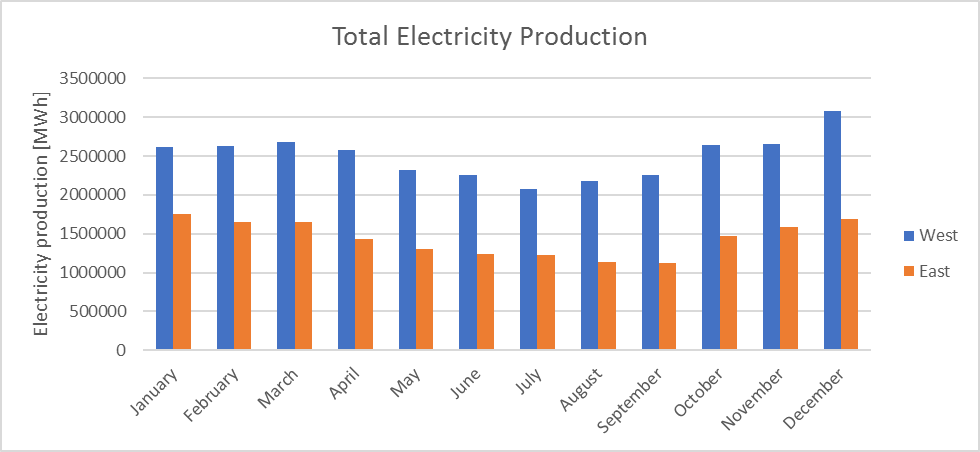
The results follow the same procedure as the article, with the initial results covering the data from the measured electrical production of the power grid, in order to determine if there are significant changes in the impact scores pr. kWh throughout the year, and if so to quantify these.

Figure 1: Total monthly electricity production for east and west Denmark

The results for the eastern electrical grid are not entirely similar to the western grid, while the overall trend is the same with the scores in general being lower during the summer, the period in which the results are significantly lower is longer than it is in the west. In the east the average impact pr. kWh is markedly lower from May to September, while this reduction in the west is limited to June and July. This is caused by two factors, firstly the fact that the grids are inherently different in their composition, secondly they differ in scale. West Denmark produces on average 75% more electricity than the east, and in some months up to twice the amount. Figure 1 shows the total electricity produced in east and west Denmark, outlines the difference on a monthly basis, and shows that the western electrical grid supplies between 49-101% more electricity than the east.

This difference in scale has great effect on the overall impact scores contributed to the electricity production, given the fact that the composition of the sources supplying the electricity varies as the demand scales up. As outlined in the article, the total electricity potential supplied from renewable sources within the current electrical infrastructure is limited, and any amount of electricity produced in excess of what the renewable sources are capable of will be supplied from import or non-renewable sources.

Table 1: Deviations in midpoint scores pr. kWh on a monthly basis relative to the yearly average, calculated from the hourly data resolution, eastern Denmark

|  | January | February | March | April | May | June | July | August | September | October | November | December |
| --- | --- | --- | --- | --- | --- | --- | --- | --- | --- | --- | --- | --- |
| Fossil depletion | 120% | 125% | 138% | 117% | 79% | 80% | 74% | 69% | 68% | 108% | 104% | 118% |
| Marine eutrophication | 111% | 118% | 137% | 119% | 81% | 87% | 76% | 71% | 63% | 114% | 101% | 122% |
| Terrestrial acidification | 138% | 142% | 145% | 116% | 72% | 66% | 68% | 62% | 73% | 98% | 108% | 112% |
| Ionizing radiation | 70% | 73% | 109% | 112% | 107% | 120% | 102% | 97% | 66% | 127% | 93% | 123% |
| Freshwater ecotoxicity | 80% | 93% | 116% | 118% | 97% | 113% | 88% | 88% | 71% | 121% | 92% | 121% |
| Photochemical oxidant formation | 135% | 138% | 143% | 116% | 74% | 68% | 70% | 64% | 73% | 99% | 108% | 112% |
| Terrestrial ecotoxicity | 121% | 123% | 134% | 115% | 92% | 81% | 81% | 73% | 81% | 95% | 102% | 102% |
| Marine ecotoxicity | 82% | 94% | 118% | 118% | 96% | 111% | 87% | 87% | 70% | 121% | 93% | 121% |
| Climate Change | 124% | 129% | 140% | 117% | 75% | 77% | 73% | 68% | 68% | 107% | 104% | 118% |
| Particulate matter formation | 131% | 135% | 139% | 115% | 76% | 72% | 73% | 68% | 76% | 99% | 106% | 111% |
| Human toxicity | 97% | 106% | 132% | 121% | 84% | 98% | 80% | 77% | 60% | 121% | 97% | 126% |
| Metal depletion | 86% | 95% | 102% | 108% | 103% | 110% | 94% | 96% | 93% | 110% | 96% | 109% |
| Natural land transformation | 117% | 116% | 124% | 108% | 91% | 83% | 87% | 81% | 83% | 99% | 105% | 106% |
| Urban land occupation | 147% | 151% | 149% | 117% | 66% | 59% | 63% | 58% | 74% | 95% | 110% | 112% |
| Water depletion | 77% | 69% | 82% | 93% | 119% | 122% | 127% | 124% | 107% | 101% | 91% | 89% |
| Freshwater eutrophication | 86% | 96% | 131% | 123% | 86% | 107% | 82% | 79% | 52% | 130% | 94% | 134% |
| Ozone depletion | 130% | 132% | 140% | 115% | 79% | 72% | 73% | 66% | 73% | 100% | 108% | 112% |
| Agricultural land occupation | 153% | 156% | 153% | 117% | 64% | 54% | 61% | 55% | 73% | 92% | 112% | 111% |

Table 1 above shows the monthly deviations in midpoint scores from the yearly averages for the electrical grid of eastern Denmark, and shows the difference in each impact category from the monthly results to the yearly averages. Scores that are significantly lower than the average are denoted by a green fill, while scores that are significantly higher are denoted by orange/red fills, scores marked with yellow are close to the yearly average. The corresponding table for the west is placed in the article.

Table 2: Deviations in endpoint scores pr. kWh on a monthly basis relative to the yearly average, calculated from the hourly data resolution, eastern Denmark

|  | January | February | March | April | May | June | July | August | September | October | November | December |
| --- | --- | --- | --- | --- | --- | --- | --- | --- | --- | --- | --- | --- |
| Ecosystems | | | | | | | | | | | | |
| Agricultural land occupation | 153% | 156% | 152% | 117% | 64% | 54% | 61% | 55% | 73% | 92% | 112% | 112% |
| Climate Change | 124% | 129% | 140% | 118% | 76% | 77% | 73% | 68% | 68% | 107% | 104% | 117% |
| Freshwater ecotoxicity | 81% | 93% | 117% | 119% | 98% | 113% | 88% | 88% | 71% | 122% | 92% | 118% |
| Freshwater eutrophication | 86% | 97% | 132% | 123% | 87% | 107% | 82% | 80% | 52% | 130% | 94% | 130% |
| Marine ecotoxicity | 83% | 95% | 118% | 119% | 97% | 112% | 88% | 88% | 71% | 121% | 93% | 118% |
| Natural land transformation | 107% | 103% | 112% | 103% | 99% | 93% | 98% | 92% | 90% | 99% | 102% | 101% |
| Terrestrial acidification | 138% | 142% | 145% | 116% | 72% | 66% | 68% | 62% | 73% | 98% | 108% | 113% |
| Terrestrial ecotoxicity | 121% | 123% | 134% | 115% | 92% | 81% | 81% | 73% | 81% | 95% | 102% | 103% |
| Urban land occupation | 146% | 151% | 149% | 116% | 66% | 59% | 63% | 58% | 74% | 95% | 110% | 113% |
| Human Health | | | | | | | | | | | | |
| Climate Change | 124% | 129% | 140% | 118% | 76% | 77% | 73% | 68% | 68% | 107% | 104% | 117% |
| Human toxicity | 97% | 107% | 133% | 121% | 84% | 99% | 80% | 77% | 60% | 121% | 97% | 123% |
| Ionizing radiation | 70% | 73% | 110% | 113% | 107% | 121% | 102% | 97% | 67% | 127% | 93% | 120% |
| Ozone depletion | 127% | 129% | 138% | 114% | 82% | 74% | 75% | 67% | 74% | 100% | 107% | 112% |
| Particulate matter formation | 132% | 135% | 139% | 114% | 76% | 71% | 73% | 68% | 76% | 99% | 106% | 111% |
| Photochemical oxidant formation | 137% | 140% | 144% | 116% | 73% | 67% | 69% | 63% | 73% | 98% | 108% | 113% |
| Resources | | | | | | | | | | | | |
| Fossil depletion | 120% | 125% | 138% | 117% | 79% | 80% | 74% | 69% | 68% | 108% | 104% | 117% |
| Metal depletion | 86% | 95% | 102% | 108% | 103% | 110% | 94% | 96% | 93% | 110% | 96% | 107% |

Tables 2 and 3 show the deviations for the endpoint scores calculated using hourly data resolution. The difference between the monthly deviations is minimal between midpoints and endpoints, on average 1.5% and maximally 2.7%. This means that the monthly deviations are not identical depending on whether midpoint scores or endpoint scores are calculated. Table 2 shows the eastern grid and table 3 the western grid.

Table 3: Deviations in endpoint scores pr. kWh on a monthly basis relative to the yearly average, calculated from the hourly data resolution, western Denmark

|  | January | February | March | April | May | June | July | August | September | October | November | December |
| --- | --- | --- | --- | --- | --- | --- | --- | --- | --- | --- | --- | --- |
| Ecosystems | | | | | | | | | | | | |
| Agricultural land occupation | 153% | 143% | 138% | 109% | 98% | 38% | 43% | 65% | 91% | 81% | 110% | 130% |
| Climate Change | 120% | 125% | 120% | 106% | 96% | 57% | 51% | 99% | 104% | 100% | 103% | 118% |
| Freshwater ecotoxicity | 88% | 108% | 103% | 108% | 99% | 92% | 77% | 120% | 103% | 117% | 96% | 89% |
| Freshwater eutrophication | 89% | 112% | 105% | 103% | 92% | 71% | 52% | 133% | 117% | 122% | 97% | 106% |
| Marine ecotoxicity | 89% | 108% | 104% | 108% | 99% | 91% | 75% | 120% | 103% | 116% | 96% | 90% |
| Natural land transformation | 107% | 100% | 106% | 97% | 100% | 84% | 91% | 103% | 106% | 95% | 105% | 106% |
| Terrestrial acidification | 136% | 132% | 129% | 108% | 98% | 52% | 53% | 81% | 95% | 90% | 107% | 120% |
| Terrestrial ecotoxicity | 118% | 115% | 121% | 109% | 108% | 76% | 80% | 91% | 93% | 91% | 104% | 95% |
| Urban land occupation | 145% | 140% | 134% | 109% | 98% | 45% | 46% | 71% | 92% | 85% | 108% | 126% |
| Human Health | | | | | | | | | | | | |
| Climate Change | 120% | 125% | 120% | 106% | 96% | 57% | 51% | 99% | 104% | 100% | 103% | 118% |
| Human toxicity | 97% | 115% | 109% | 105% | 95% | 70% | 56% | 122% | 112% | 115% | 97% | 107% |
| Ionizing radiation | 79% | 96% | 97% | 98% | 94% | 89% | 77% | 137% | 116% | 121% | 100% | 95% |
| Ozone depletion | 125% | 122% | 123% | 106% | 98% | 64% | 65% | 90% | 95% | 95% | 109% | 107% |
| Particulate matter formation | 129% | 127% | 124% | 107% | 99% | 59% | 60% | 86% | 97% | 92% | 106% | 116% |
| Photochemical oxidant formation | 134% | 131% | 127% | 107% | 98% | 53% | 54% | 82% | 96% | 91% | 107% | 119% |
| Resources | | | | | | | | | | | | |
| Fossil depletion | 117% | 123% | 119% | 105% | 96% | 61% | 56% | 101% | 103% | 102% | 104% | 113% |
| Metal depletion | 93% | 105% | 102% | 107% | 101% | 105% | 95% | 106% | 94% | 111% | 99% | 83% |

The substantial difference in overall electricity demand between east and west Denmark directly affects the average environmental impact caused by the electricity production. Table 4 shows the yearly averages for the impact scores caused by the electrical grid pr. kWh, calculated from an hourly grid resolution, and averaged to yearly values. The table also shows the difference for each impact category between the eastern and western grids, as a percentage. The results show that the differences in the electrical grids directly influences the resulting environmental impacts. The difference between east and west is between -20% and 37%, depending on the impact category, where east has higher impact on water depletion and agricultural land occupation, while west has higher impact on freshwater and marine ecotoxicity, to name a few.

Table 4: Midpoint impact scores pr. kWh averaged over the year, and difference between east and west in percentage values

| impact category | East | West | unit | Difference |
| --- | --- | --- | --- | --- |
| Fossil depletion | 5,22E-02 | 5,40E-02 | kg oil eq /kWh | 6,3% |
| Marine eutrophication | 4,74E-05 | 5,20E-05 | kg N eq /kWh | 13,6% |
| Terrestrial acidification | 8,33E-04 | 7,76E-04 | kg SO2 eq /kWh | -5,1% |
| Ionizing radiation | 1,82E-02 | 2,31E-02 | kg U235 eq /kWh | 32,5% |
| Freshwater ecotoxicity | 2,69E-03 | 3,61E-03 | kg 1,4-DB eq /kWh | 37,1% |
| Photochemical oxidant formation | 5,25E-04 | 5,29E-04 | kg NMVOC /kWh | 2,3% |
| Terrestrial ecotoxicity | 2,53E-05 | 2,74E-05 | kg 1,4-DB eq /kWh | 9,6% |
| Marine ecotoxicity | 2,56E-03 | 3,39E-03 | kg 1,4-DB eq /kWh | 35,3% |
| Climate Change | 2,07E-01 | 2,05E-01 | kg CO2 eq /kWh | 1,6% |
| Particulate matter formation | 2,68E-04 | 2,56E-04 | kg PM10 eq /kWh | -3,2% |
| Human toxicity | 8,29E-02 | 9,57E-02 | kg 1,4-DB eq /kWh | 20,2% |
| Metal depletion | 5,94E-03 | 7,76E-03 | kg Fe eq /kWh | 31,0% |
| Natural land transformation | 2,88E-05 | 2,86E-05 | m2 /kWh | -0,2% |
| Urban land occupation | 4,68E-03 | 4,11E-03 | m2*a /kWh | -10,5% |
| Water depletion | 7,81E-01 | 6,28E-01 | m3 /kWh | -20,0% |
| Freshwater eutrophication | 1,07E-04 | 1,31E-04 | kg P eq /kWh | 30,8% |
| Ozone depletion | 2,59E-08 | 2,65E-08 | kg CFC-11 eq /kWh | 4,4% |
| Agricultural land occupation | 3,63E-01 | 3,07E-01 | m2*a /kWh | -14,2% |

Figure 2 shows the average electrical grid composition for east and west Denmark, while this composition is simplified to show only the yearly average, it shows that the two power grids are distinct from one another and that the environmental performance of a building may depend on whether it is in east or west Denmark.

|  | January | February | March | April | May | June | July | August | September | October | November | December | unit |
| --- | --- | --- | --- | --- | --- | --- | --- | --- | --- | --- | --- | --- | --- |
| West | 2,62 | 2,63 | 2,69 | 2,58 | 2,32 | 2,25 | 2,07 | 2,17 | 2,25 | 2,64 | 2,65 | 3,09 | TWh |
| East | 1,76 | 1,66 | 1,65 | 1,44 | 1,31 | 1,24 | 1,23 | 1,14 | 1,12 | 1,48 | 1,59 | 1,69 | TWh |
| Difference | 49% | 59% | 63% | 80% | 78% | 82% | 68% | 91% | 101% | 79% | 67% | 82% | % |

Table 5: Average monthly electricity production in TWh in east and west Denmark, and difference between the two as a percentage


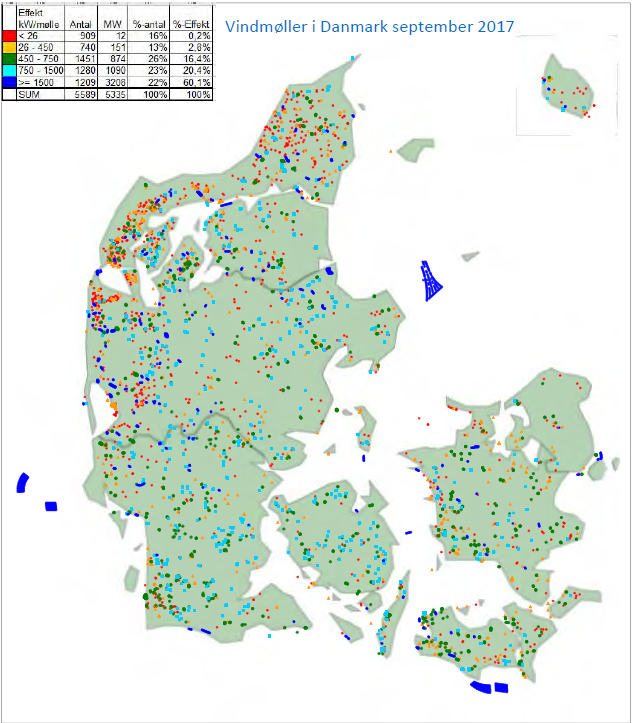

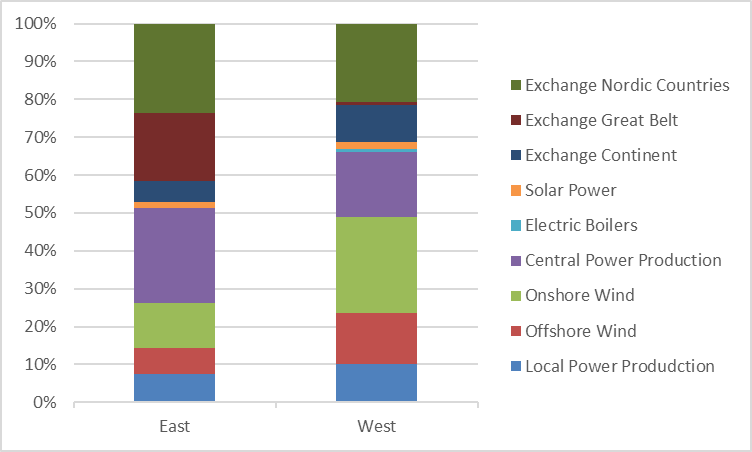
On average 47.1% of the electricity is imported for east Denmark, while the west only imports 31.3%, however 38% the imported electricity in the east is imported from west Denmark. Another key factor is wind power, which contributes 39% of the total western electricity production, while only 18.5% of the eastern grid. This is due to the majority of the expansion of the windmill construction taking place in western Denmark, as seen in figure 3.

Figure 2: Average grid composition for the year 2017

The impact scores pr. kWh displayed in table 4 show that the impact scores for the western electricity grid are over 30% higher than the eastern grid in five categories; Ionizing radiation, freshwater ecotoxicity, marine ecotoxicity, metal depletion, and lastly freshwater eutrophication. Of these five categories excluding metal depletion, the remaining four are the ones where the highest difference between the dynamic grids and reference grid are found. It is therefore very likely that the factors causing this difference between the eastern and western grids are also responsible for the substantial difference in the reference grid, only to a lesser degree.

Figure 3: Windmills in Denmark - September 2017. Source: http://dkvind.dk/html/nogletal/pdf/moeller_dk_2017.pdf

## 2.2 Building electricity results

Table 4 shows the 18 buildings selected for the study, with their corresponding areas, years of construction, age, and total yearly electricity consumption in kWh. The buildings were selected in order to have diversity both in age and total area, so that the effect of these two parameters on the EBP can be determined.

Table 6: Building data set

| Building | Total Area [m2] | Construction year | Age [years] | Electricity [kWh] |
| --- | --- | --- | --- | --- |
| W1 | 2300 | 1980 | 37 | 134719,7 |
| W2 | 3461 | 1884 | 133 |  |
| W3 | 2066 | 1994 | 23 | 100910,93 |
| W4 | 5466 | 1968 | 49 | 142618,26 |
| W5 | 2162 | 1837 | 180 | 83305,8 |
| W6 | 1835 | 1977 | 40 | 147320,76 |
| W7 | 7560 | 1978 | 39 | 292964,91 |
| W8 | 19604 | 1969 | 48 | 640826,95 |
| W9 | 2265 | 1887 | 130 | 65198,48 |
| E1 | 5481 | 1963 | 54 | 236837,88 |
| E2 | 9258 | 1978 | 39 | 287179,04 |
| E3 | 4330 | 1890 | 127 | 178824,01 |
| E4 | 3769 | 1854 | 163 | 114438,86 |
| E5 | 3560 | 1784 | 233 | 124632,56 |
| E6 | 5920 | 1706 | 311 | 166167,02 |
| E7 | 8543 | 1940 | 77 | 344649,4 |
| E8 | 7341 | 1798 | 219 | 183383,67 |
| E9 | 5936 | 1771 | 246 | 136792,74 |

The electricity consumption for building W2 was only available as a monthly resolution, while the data for the remaining buildings was in an hourly resolution, as such building W2 was excluded from the comparison.

Table 7: Age groups for buildings, divided into three groups

| Zone | Age group I | Age group II | Age group III | unit |
| --- | --- | --- | --- | --- |
| West | 23-39 | 40-49 | 133-180 | years |
| East | 39-77 | 127-219 | 233-311 | years |

The buildings were divided into three groups based on their ages, as displayed in table 5 above, with the brackets being determined by the ages present in the building data set, which is why the intervals differ from east to west because of the eastern buildings being significantly older.


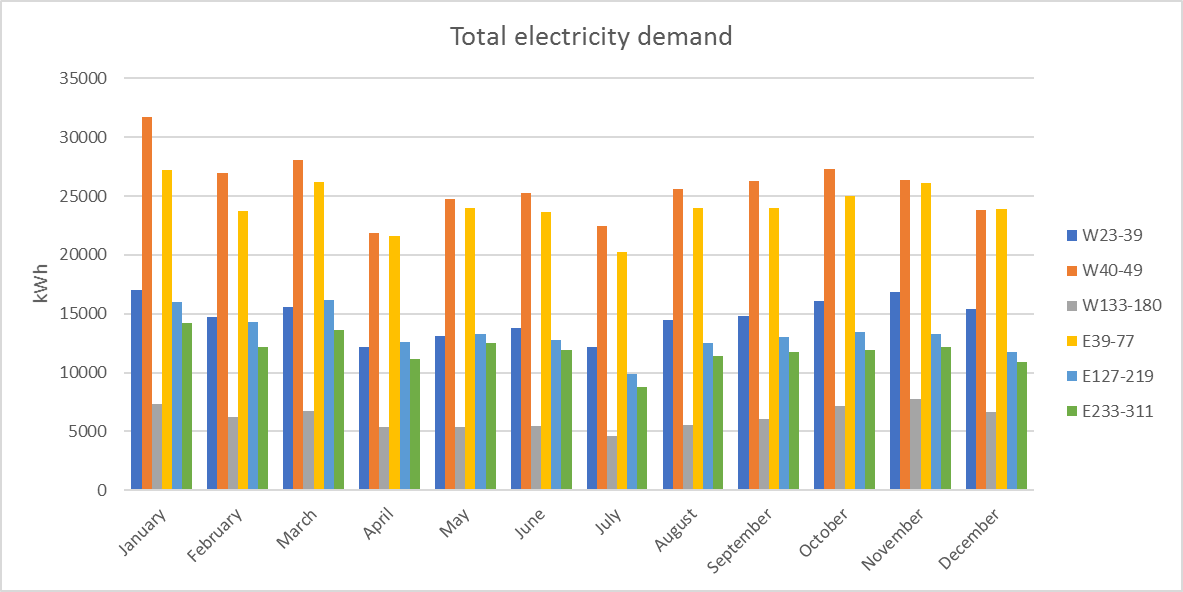
The total electricity consumption for each building age group is displayed in figure 4 above, and shows the average electricity demand within each bracket for each month in kWh. The data shows that in general the newer buildings use more electricity than their older counterparts, and that the lowest consumption is seen for the oldest buildings. The largest electricity consumption can be seen for western age group II and eastern age group I.

Figure 4: Total electricity consumption for all buildings, divided into age brackets

Figure 5 below shows the electricity demand pr. m2, averaged within each age group and displayed on a monthly basis. This figure shows a different picture, with the eastern age bracket I no longer being one of the most demanding, and instead it is age groups I & II for west that have the largest electricity consumption/m2. The general trend is a higher electricity demand pr. m2 for the buildings in western Denmark, with the average kWh/year/m2 for western buildings being 44.1 and 33 for eastern buildings, across all age brackets. This is due to the overall electricity demand being very similar between east and west, but the western buildings are on average 4565 m2 while the eastern are averagely 6015 m2, which leads to higher consumption/m2 for the west.

The basis of comparison between the buildings is the environmental impact pr. m2, which is directly tied to the electricity consumption pr. m2. As such there will be a direct correlation between the data
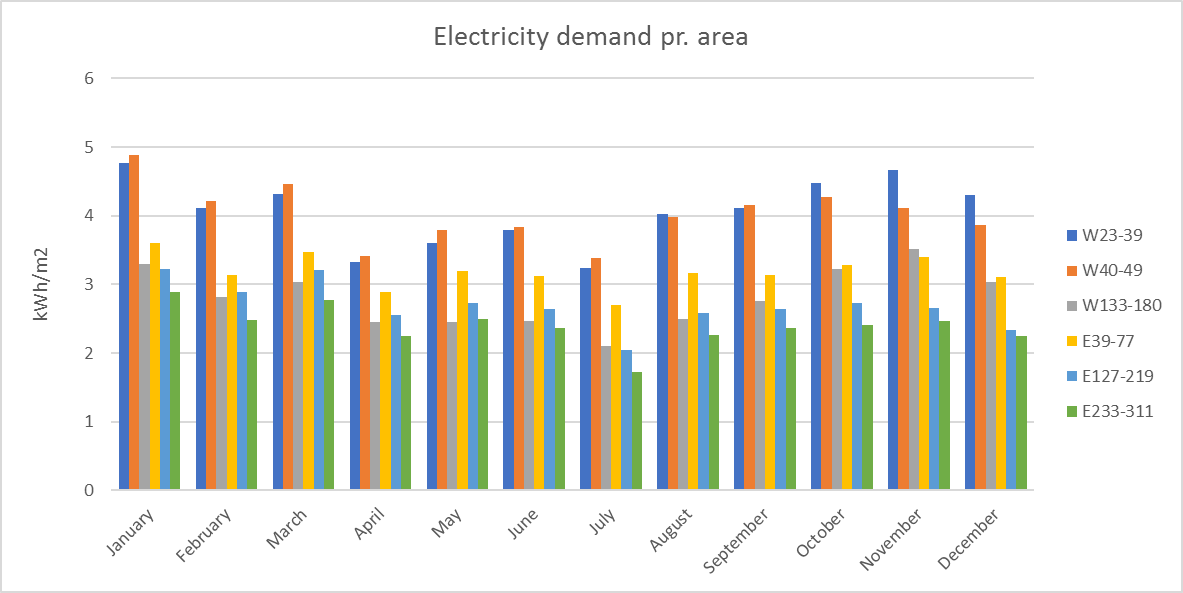
displayed in figure 5 and the overall EBP, within each section of Denmark. The differences in electrical grid composition may bridge the gap between the western and eastern buildings, however only for certain impact categories where the western power grid has on average lower scores pr. kWh, the other categories where the impacts are higher for the western grid will only exacerbate the difference between west and east.

Figure 5: Electricity demand pr. m2 for all buildings, divided into age brackets

## 2.3 Electrical grid compositions

In order for the comparison between the dynamic high-resolution electrical grids to the static reference grids from the database to be fair, it is important to consider the composition of both grids, in order to ascertain if the systems are similar enough to justify the comparison, as outlined in ISO 14044:

“*Systems shall be compared using the same functional unit and equivalent methodological considerations such as performance, system boundaries, data quality, allocation procedures, decision rules on evaluating inputs, and outputs and impact assessment. Any differences between systems regarding these parameters shall be identified and reported. If the study is intended to be used for a comparative assertion intended to be disclosed to the public, interested parties shall conduct this evaluation as a critical review*” (ISO 14044, 2006)

In the context of this study the most critical part is the data quality, and as such it is critical to account for the processes included in either type of electrical grid composition, to ensure that both are representative of the actual grid, and any comparison between the two is accurate.

### 2.3.1 Dynamic high-resolution grid

The dynamic high-resolution grids are determined using various resolutions, for all resolutions the basis reference impact scores pr. kWh for each source are unchanged, and are found using processes in the Ecoinvent database.

For each energy source the environmental impacts caused by the production of 1 kWh are found, using existing processes in the Ecoinvent database, as they detail the production of electricity for Denmark. Once the reference impact score values are found for each energy source contributing to the electrical grid, the percentage of each source at any given hour is found, and the total environmental impact for a kWh at the chosen time step is determined by summarizing the relative contribution of each source for all impact categories.

### 2.3.2 Static reference grid

The static reference grid is calculated using the process *market for electricity, low voltage | electricity, low voltage | APOS, S*. This process is comprised of a number of other processes, minor contributions from the distribution network, solar panels, and sulfur hexafluoride. The main component links to a process for the transformation from medium to low voltage, which mainly includes the process for medium voltage electricity production, which through the same procedure ends up linking to the production of high-voltage electricity in Denmark. Along the way from low to high voltage there are transmission and transformation losses, which means in order for the grid to supply 1 kWh of low voltage electricity, more than 1 kWh of high voltage electricity has to be produced.

The process *market for electricity, high voltage* is comprised of a number of processes each detailing the electricity production from a given energy source. The processes contributing to the production of the high voltage electricity are the same processes used to construct the high-resolution grid, with the exception of the processes concerning the transmission network construction, which are not included in the dynamic grids. These are however minor contributions of 6.58E-9 km and 3.17E-10 km respectively. And the contributions to the impact scores from the transmission network constructions pr. kWh are on average below 1%, with exception for natural land transformation to which they contribute 4%.

The major components are as such the same in the two methods for determining the electrical grid, and the only variation being the amounts that each source contributes to the production at any given time. As well as the fact that the losses due to transmission and transformation are calculated according to the dynamic data resolution, based on the Gross and Net production, and not from static reference processes as with the reference system. As such the loss factor in the electricity production follows the same dynamic practices used on the remainder of the grid, and is calculated by the hour/day/month based on the resolution. The loss factor is then multiplied with the given electricity consumption, thereby representing the losses caused by transformation and transmission, this loss factor varies between 2-6% based on the electrical grid.

# 3 Analysis

## 3.1 Hourly grid resolution


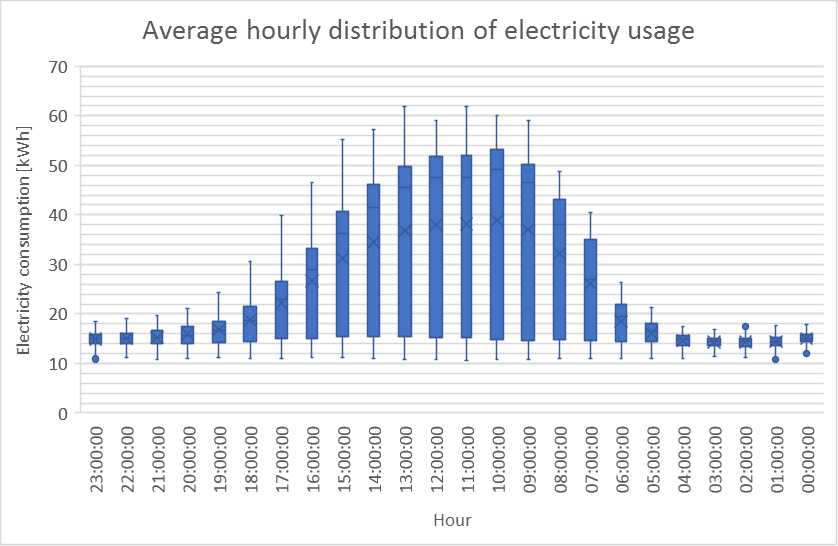
Figures 6 & 7 below show the average hourly distribution of the electrical consumption throughout the day, calculated from the total yearly electrical data from the hourly resolution, figure 6 shows eastern buildings and figure 7 shows western buildings. Eastern buildings use above average electricity between 07-16, while western buildings use above average between 07-15.


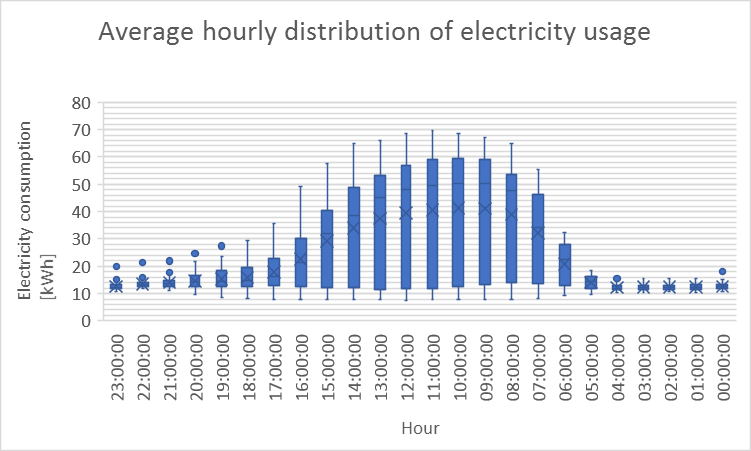


Figure 6: Average distribution of electricity usage during the day, for eastern buildings

Figure 7: Average distribution of electricity usage during the day, for western buildings

Figure 8 shows the average distribution of midpoint impact score results pr. kWh throughout the day, based on total yearly data of an hourly data resolution. The graph shows that the electricity production during a day has the highest environmental impact pr. kWh during the same intervals where the buildings consume the most electricity, in the east the impacts pr. kWh are higher than the average between 10-14 while they are higher between 7-15 for western Denmark. Both intervals overlap with the peak electricity consumption times for the buildings.

The hourly resolution building electricity consumption shows that the eastern buildings use on average 64% of their total electricity between 7-17, this interval is responsible for 67% of the total electricity demand for the western buildings. The hourly resolutions are able to account for the difference between the active and inactive periods of the buildings, which due to being commercial buildings are only in use during business hours, and are not in use during the night and over weekends, during these times the electricity consumption is reduced to a baseline value.


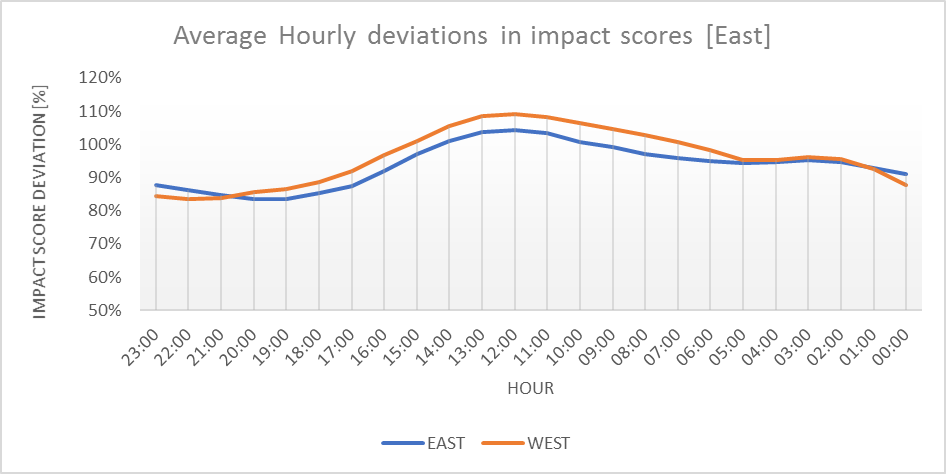


Figure 8: Deviation in midpoint impact scores pr. kWh from the average, on an hourly basis. Eastern Denmark in blue, and western Denmark in orange

## 3.2 Daily grid resolution


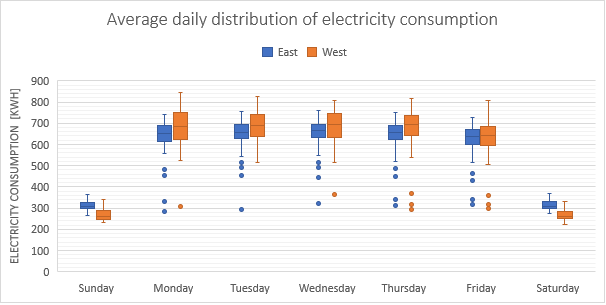


Figure 9: Average daily distribution of electricity consumption between weekdays, east in blue and west in orange

Figure 9 above shows the average distribution of the building electricity consumption during the week, calculated from the daily resolution electricity consumption, averaged over the whole year. The graph shows the daily consumption in the east in blue and west in orange. While the western buildings require more electricity in the work days when compared to the eastern buildings they consume less electricity over the weekend.

While the daily grid resolutions cannot account for the peak times for electricity demand throughout the day, or the difference between day and night, they do include the distinction between active and inactive days, such as workdays and weekends. The results show that eastern buildings use on average two times as much electricity during the active days of the week when compared to ‘inactive’ days of the weekend, for the western buildings the active days use 2.5 times as much electricity compared to the weekend.

This is an important factor to consider, since reference grids or grids of a lower resolution assume all days to be equal in terms of electricity usage, which is clearly not the case. When combined with the daily variations of the impact score results resulting from the electricity production, the daily resolution grid data proves to be an acceptable surrogate for an hourly grid resolution, should data with an hourly resolution be unavailable.

## 3.3 Monthly grid resolution

Figure 10: Average monthly electricity usage [kWh/m2] and deviation in impact score [%], western Denmark

Figure 11: Average monthly electricity usage [kWh/m2] and deviation in impact score [%], eastern Denmark

Figures 10 & 11 above show the average monthly electricity demand pr. m2 for the buildings, figure 10 shows the three eastern age groups and figure 11 the three western age groups, both graphs include the corresponding monthly deviations from the yearly average for the total impact scores pr. kWh, for their corresponding sections of Denmark.

The graphs show that the older buildings in general require less electricity, with the exception for western buildings in May and July, where the second age group has a higher electricity consumption than the first age group, otherwise the demand decreases as the age increases.

Furthermore the monthly resolution results show a similarity between the electricity usage and deviation in impact score results, that are both reduced during the summer months, and the two are correlated throughout the year.

## 3.4 Comparison

### 3.4.1 Midpoint results

Table 8: Average yearly midpoint scores pr. kWh for western Denmark, calculated from the hourly grid resolution. And compared to the scores from the other resolutions, as well as the reference grid

| Resolution Comparisons | unit | Hourly | Daily | Monthly | Yearly | Reference |
| --- | --- | --- | --- | --- | --- | --- |
| Fossil depletion | kg oil eq /kWh | 5,40E-02 | 100,03% | 99,03% | 108,99% | 177,80% |
| Marine eutrophication | kg N eq /kWh | 5,20E-05 | 100,17% | 99,47% | 116,69% | 134,07% |
| Terrestrial acidification | kg SO2 eq /kWh | 7,76E-04 | 99,91% | 98,52% | 91,31% | 155,96% |
| Ionizing radiation | kg U235 eq /kWh | 2,31E-02 | 100,15% | 99,60% | 134,54% | 503,59% |
| Freshwater ecotoxicity | kg 1,4-DB eq /kWh | 3,61E-03 | 100,68% | 101,37% | 115,85% | 569,38% |
| Photochemical oxidant formation | kg NMVOC /kWh | 5,29E-04 | 99,91% | 98,58% | 92,49% | 132,37% |
| Terrestrial ecotoxicity | kg 1,4-DB eq /kWh | 2,74E-05 | 100,39% | 99,01% | 84,71% | 201,89% |
| Marine ecotoxicity | kg 1,4-DB eq /kWh | 3,39E-03 | 100,66% | 101,26% | 116,09% | 535,26% |
| Climate Change | kg CO2 eq /kWh | 2,05E-01 | 100,07% | 99,04% | 109,37% | 184,64% |
| Particulate matter formation | kg PM10 eq /kWh | 2,56E-04 | 93,35% | 92,00% | 94,05% | 148,66% |
| Human toxicity | kg 1,4-DB eq /kWh | 9,57E-02 | 100,41% | 100,12% | 127,74% | 150,48% |
| Metal depletion | kg Fe eq /kWh | 7,76E-03 | 100,35% | 101,31% | 98,29% | 220,92% |
| Natural land transformation | m2 /kWh | 2,86E-05 | 99,76% | 98,25% | 97,71% | 184,48% |
| Urban land occupation | m2*a /kWh | 4,11E-03 | 99,89% | 98,55% | 85,90% | 115,75% |
| Water depletion | m3 /kWh | 6,28E-01 | 100,26% | 98,47% | 134,16% | 668,88% |
| Freshwater eutrophication | kg P eq /kWh | 1,31E-04 | 100,41% | 100,22% | 138,93% | 121,90% |
| Ozone depletion | kg CFC-11 eq /kWh | 2,65E-08 | 99,83% | 98,46% | 93,07% | 182,88% |
| Agricultural land occupation | m2*a /kWh | 3,07E-01 | 99,75% | 97,93% | 80,96% | 75,06% |

Table 7 above shows the midpoint impact scores pr. kWh for western Denmark, calculated from the hourly grid resolution and averaged for the year in the first column. The remaining columns show the hourly resolution scores compared to the scores of the other grid resolutions, as well as the scores of the reference grid system. Percentages over 100% indicate that the impact score from the grid in question is higher than the corresponding hourly score, as do percentages under 100% indicate scores that are lower.

The comparison shows that the difference in the yearly average impact scores results between the hourly resolution grid and the daily resolution grid, with the exception for particulate matter formation, never exceeds 1%. As such the hourly and daily resolution grids are nearly equivalent as far as the electrical grid is concerned, with regard to the EBP the differences become clearer.

The comparison between the hourly resolution grid and the monthly resolution grid shows only slightly higher differences than the previous comparison, with the deviations based on the impact category are up to 2%, with particulate matter formation again being an outlier.

The comparison between the hourly and yearly grid resolutions are more substantial, where the impact scores for the yearly resolution range between 81% and 139% of their hourly resolution counterparts, and these two grids would produce markedly different environmental performances when used in a building LCA. The comparison between the resolutions show that while there are minor differences between the hourly, daily, and monthly resolutions, the yearly resolution results in the largest degree of change in impact score results, and as such the inclusion of the monthly variations of the electrical grid in the dynamic grid modelling prove to be the most impactful on the overall impacts associated with the electricity production.

The comparison between the hourly resolution electrical grid and the reference grid are the most significant, with the reference grid scores ranging between 75% and 669% or the hourly resolution scores, with the only category scoring lower for the reference grid being agricultural land occupation, while all other scores are significantly higher. The categories where the difference exceeds 250% are likely due to methodological differences in the calculation of transmission and transformation losses, where the reference grid includes certain processes attributed to the construction of the grid infrastructure that are not present in the dynamic calculations, as well as the dynamic modelling practices used in the loss factor calculations for the dynamic grids, whose counterparts are static in the reference grid.

Table 9: Average yearly midpoint scores pr. kWh for eastern Denmark, calculated from the hourly grid resolution. And compared to the scores from the other resolutions, as well as the reference grid

| Resolution Comparisons | unit | Hourly | Daily | Monthly | Yearly | Reference |
| --- | --- | --- | --- | --- | --- | --- |
| Fossil depletion | kg oil eq /kWh | 5,22E-02 | 99,22% | 98,74% | 119,99% | 184,13% |
| Marine eutrophication | kg N eq /kWh | 4,74E-05 | 99,12% | 98,58% | 110,87% | 147,33% |
| Terrestrial acidification | kg SO2 eq /kWh | 8,33E-04 | 99,41% | 99,10% | 138,30% | 145,21% |
| Ionizing radiation | kg U235 eq /kWh | 1,82E-02 | 99,16% | 98,28% | 69,69% | 641,58% |
| Freshwater ecotoxicity | kg 1,4-DB eq /kWh | 2,69E-03 | 99,50% | 99,32% | 80,47% | 764,39% |
| Photochemical oxidant formation | kg NMVOC /kWh | 5,25E-04 | 106,58% | 106,25% | 134,78% | 133,28% |
| Terrestrial ecotoxicity | kg 1,4-DB eq /kWh | 2,53E-05 | 100,06% | 99,67% | 120,69% | 218,77% |
| Marine ecotoxicity | kg 1,4-DB eq /kWh | 2,56E-03 | 99,48% | 99,27% | 82,27% | 709,12% |
| Climate Change | kg CO2 eq /kWh | 2,07E-01 | 99,23% | 98,77% | 123,65% | 182,56% |
| Particulate matter formation | kg PM10 eq /kWh | 2,68E-04 | 95,55% | 95,22% | 131,46% | 141,60% |
| Human toxicity | kg 1,4-DB eq /kWh | 8,29E-02 | 99,06% | 98,46% | 97,13% | 173,59% |
| Metal depletion | kg Fe eq /kWh | 5,94E-03 | 99,88% | 100,15% | 85,78% | 288,72% |
| Natural land transformation | m2 /kWh | 2,88E-05 | 99,66% | 99,21% | 117,36% | 183,06% |
| Urban land occupation | m2*a /kWh | 4,68E-03 | 99,39% | 99,20% | 146,68% | 101,79% |
| Water depletion | m3 /kWh | 7,81E-01 | 100,46% | 99,74% | 77,47% | 537,24% |
| Freshwater eutrophication | kg P eq /kWh | 1,07E-04 | 98,78% | 98,00% | 85,60% | 149,44% |
| Ozone depletion | kg CFC-11 eq /kWh | 2,59E-08 | 99,41% | 99,02% | 129,76% | 187,31% |
| Agricultural land occupation | m2*a /kWh | 3,63E-01 | 99,39% | 99,14% | 152,76% | 63,49% |

Table 8 shows the same comparison but for the eastern Danish electrical grid, that in general shows the same trends as the western electrical grid, with the exception of photochemical oxidant formation, that in the western grid was similar to the other categories, that in the eastern grid returns higher results when lowering the grid resolution.

Otherwise the tendencies are identical to those of the western grid, with differences from hourly resolution to daily/monthly being minor and the yearly resolution sees spikes in differences. And lastly the reference grid which also for the eastern comparison is extremely different to the dynamic grids.

### 3.4.2 Endpoint results

Table 10: Average yearly endpoint scores pr. kWh for western Denmark, calculated from the hourly grid resolution. And compared to the scores from the other resolutions, as well as the reference grid

| Resolution Comparisons | unit | Hourly | Daily | Monthly | Yearly | Reference |
| --- | --- | --- | --- | --- | --- | --- |
| Ecosystems | Species/year /kWh | 5,70E-09 | 98,3% | 96,8% | 88,8% | 112,2% |
| Human Health | DALY /kWh | 4,23E-07 | 98,0% | 97,1% | 109,4% | 173,3% |
| Resources | $ /kWh | 9,50E-03 | 100,0% | 99,1% | 108,3% | 180,1% |

Table 11: Average yearly endpoint scores pr. kWh for eastern Denmark, calculated from the hourly grid resolution. And compared to the scores from the other resolutions, as well as the reference grid

| Resolution Comparisons | unit | Hourly | Daily | Monthly | Yearly | Reference |
| --- | --- | --- | --- | --- | --- | --- |
| Ecosystems | Species/year /kWh | 6,33E-09 | 96,8% | 97,5% | 93,5% | 101,0% |
| Human Health | DALY /kWh | 4,18E-07 | 94,1% | 97,3% | 108,4% | 175,2% |
| Resources | $ /kWh | 9,05E-03 | 95,8% | 98,8% | 108,1% | 189,1% |

Tables 9 & 10 show the endpoint impact scores pr. kWh for western and eastern Denmark, calculated from the hourly grid resolution and averaged for the year in the first column. The remaining columns show the hourly resolution scores compared to the scores of the other grid resolutions, as well as the scores of the reference grid system. The same procedure as with the midpoint scores.

While the method is identical to the one used for the midpoint calculations, the results from the comparison between the grid resolutions differs from the midpoints to the endpoints. Where the difference between the hourly and daily grid resolutions has minor impact on the midpoint impacts, the endpoint scores are significantly different, and the impact scores pr. kWh are notably different even between the hourly and daily grid resolutions, up to 2% for the western grid and up to 6% for the eastern. The difference between hourly and monthly grid resolutions is up to 3.2% for the western grid and up to 2.7% for the eastern, and once again the yearly grid resolution is significantly different to the hourly, with results being lower for ecosystems for both west and east, by 11.2% and 6.5% respectively. The remaining two categories are substantially higher, up to 9.4% for the western grid and up to 8.4% for the eastern grid.

The reference grid once again provides impact scores that differ greatly when compared to the dynamic grids results, and are higher for every category between 12.2% and 80% for west and between 1 and 89% for east. As such the findings from the midpoint analysis remain unchanged to the endpoint analysis, and prove that the there are vast differences in the environmental performance of a building depending on the practices employed in the determination of the electrical grid, dynamic or otherwise.

### 3.4.3 Environmental performances

The total impact score results for the midpoints and endpoints are placed in the appendices, and this section instead focuses on the effects of the various grid resolutions on the EBP, and displays the relative results between the dynamic grid resolutions.

The total impact scores are calculated for every building, with the various grid and energy consumption resolutions, after which they are summed up for each month and lastly for the year as a whole. The scores are calculated pr. m2, in order to enable the comparison between the buildings, as they vary in size.

Table 12: Difference in total midpoint scores pr. m2 between hourly and daily grid resolutions

| Hourly-Daily | W-I | W-II | W-III | E-I | E-II | E-III |
| --- | --- | --- | --- | --- | --- | --- |
| Fossil depletion | 100,8% | 98,9% | 101,2% | 100,4% | 100,6% | 100,8% |
| Marine eutrophication | 100,7% | 98,9% | 101,4% | 100,2% | 100,4% | 100,8% |
| Terrestrial acidification | 100,4% | 98,5% | 100,6% | 99,8% | 100,1% | 100,2% |
| Ionizing radiation | 101,4% | 99,2% | 102,0% | 101,9% | 101,8% | 102,5% |
| Freshwater ecotoxicity | 99,1% | 97,8% | 100,4% | 99,9% | 99,9% | 100,9% |
| Photochemical oxidant formation | 100,5% | 98,6% | 100,7% | 107,3% | 107,6% | 107,6% |
| Terrestrial ecotoxicity | 95,9% | 95,4% | 97,4% | 97,3% | 97,6% | 98,2% |
| Marine ecotoxicity | 99,2% | 97,9% | 100,5% | 99,8% | 99,8% | 100,9% |
| Climate Change | 101,2% | 99,2% | 101,6% | 100,1% | 100,4% | 100,5% |
| Particulate matter formation | 94,3% | 92,4% | 94,7% | 95,9% | 96,2% | 96,3% |
| Human toxicity | 100,9% | 99,2% | 101,9% | 99,9% | 100,1% | 100,7% |
| Metal depletion | 100,0% | 98,1% | 100,4% | 101,8% | 101,6% | 102,3% |
| Natural land transformation | 100,7% | 98,6% | 100,7% | 101,0% | 101,1% | 101,1% |
| Urban land occupation | 100,9% | 98,9% | 101,0% | 99,9% | 100,2% | 100,1% |
| Water depletion | 105,3% | 102,7% | 105,3% | 100,5% | 100,7% | 100,7% |
| Freshwater eutrophication | 101,6% | 99,6% | 102,5% | 100,5% | 100,7% | 101,4% |
| Ozone depletion | 99,7% | 97,9% | 100,0% | 100,3% | 100,5% | 100,6% |
| Agricultural land occupation | 100,7% | 98,7% | 100,7% | 99,7% | 100,1% | 99,9% |

Table 12 above shows the difference in the impact results from the hourly and daily grid resolutions, showing whether or not the daily grid resolution results in higher or lower impact score results, for the six building groups. The impact categories with the largest difference are Terrestrial ecotoxicity and particulate matter formation, that for the daily grid are between 92-98% of their hourly calculated counterparts. The main contributors to these categories are Oil for the particulate matter formation, and waste for the terrestrial ecotoxicity, and as such these energy sources must have peak contributions during certain times, that are not as impactful in the daily resolution grid. Water depletion is up to 5,3% higher for the daily grid, but only in western Denmark due to the main contributor to this category being importing energy from the continent, which is more prevalent in the west.

Table 13: Difference in total midpoint scores pr. m2 between hourly and monthly grid resolutions

| Hourly-Monthly | W-I | W-II | W-III | E-I | E-II | E-III |
| --- | --- | --- | --- | --- | --- | --- |
| Fossil depletion | 104,3% | 101,1% | 103,5% | 102,1% | 102,4% | 102,5% |
| Marine eutrophication | 106,6% | 102,8% | 105,4% | 103,4% | 103,6% | 103,8% |
| Terrestrial acidification | 99,2% | 97,5% | 99,3% | 99,2% | 99,6% | 99,6% |
| Ionizing radiation | 112,5% | 106,8% | 110,1% | 109,0% | 108,6% | 109,2% |
| Freshwater ecotoxicity | 107,2% | 103,5% | 106,6% | 104,6% | 104,5% | 105,3% |
| Photochemical oxidant formation | 99,6% | 97,8% | 99,6% | 106,7% | 107,2% | 107,2% |
| Terrestrial ecotoxicity | 96,0% | 95,6% | 97,5% | 96,8% | 97,2% | 97,8% |
| Marine ecotoxicity | 107,2% | 103,5% | 106,6% | 104,5% | 104,4% | 105,1% |
| Climate Change | 104,1% | 101,0% | 103,3% | 101,7% | 102,0% | 102,0% |
| Particulate matter formation | 93,6% | 91,8% | 93,7% | 95,6% | 96,0% | 96,1% |
| Human toxicity | 109,8% | 105,1% | 108,2% | 105,1% | 105,2% | 105,5% |
| Metal depletion | 103,8% | 101,0% | 103,7% | 103,4% | 103,2% | 103,7% |
| Natural land transformation | 101,0% | 98,7% | 100,7% | 101,1% | 101,2% | 101,3% |
| Urban land occupation | 98,0% | 96,6% | 98,3% | 98,5% | 99,1% | 98,9% |
| Water depletion | 108,0% | 104,2% | 106,8% | 102,9% | 103,0% | 103,2% |
| Freshwater eutrophication | 113,7% | 107,7% | 111,1% | 108,0% | 107,9% | 108,4% |
| Ozone depletion | 100,0% | 98,0% | 100,0% | 100,2% | 100,5% | 100,6% |
| Agricultural land occupation | 96,5% | 95,5% | 96,9% | 97,8% | 98,4% | 98,2% |

Table 13 shows the difference between the hourly and monthly grid resolution. Here the differences in the impact results become more significant, where the monthly resolution results are up to 13,7% higher than their hourly counterparts, or up to 9% lower depending on the impact category.

The complexity of the monthly grid resolution is lower than the hourly and daily resolutions, and as such nuances in the electrical grid as well as the building energy consumption are not represented, reflected in the increased difference in the impact score results.

Since the building data is unchanged between the resolutions, the variations are caused solely by increasing the number of datapoints, and thereby the accuracy and representativeness of the assessment.

Table 14: Difference in total midpoint scores pr. m2 between hourly grid resolution and reference grid

| Hourly-Reference | W-I | W-II | W-III | E-I | E-II | E-III |
| --- | --- | --- | --- | --- | --- | --- |
| Fossil depletion | 181,6% | 176,6% | 179,3% | 188,3% | 187,3% | 187,6% |
| Marine eutrophication | 139,9% | 135,4% | 137,9% | 152,3% | 151,7% | 152,0% |
| Terrestrial acidification | 151,3% | 148,9% | 150,4% | 144,7% | 143,6% | 143,8% |
| Ionizing radiation | 559,3% | 533,1% | 548,1% | 693,9% | 695,6% | 697,3% |
| Freshwater ecotoxicity | 600,5% | 580,8% | 597,1% | 786,5% | 787,1% | 791,2% |
| Photochemical oxidant formation | 128,9% | 126,8% | 128,1% | 133,2% | 132,3% | 132,5% |
| Terrestrial ecotoxicity | 190,5% | 189,6% | 192,9% | 209,9% | 209,1% | 210,6% |
| Marine ecotoxicity | 564,1% | 545,7% | 560,8% | 729,6% | 729,9% | 733,7% |
| Climate Change | 188,0% | 182,9% | 185,6% | 186,4% | 185,3% | 185,6% |
| Particulate matter formation | 145,6% | 143,1% | 144,9% | 141,6% | 140,7% | 140,9% |
| Human toxicity | 161,8% | 155,4% | 159,0% | 182,1% | 181,7% | 182,3% |
| Metal depletion | 227,0% | 221,1% | 226,8% | 293,0% | 293,3% | 294,1% |
| Natural land transformation | 183,8% | 180,0% | 182,8% | 185,1% | 184,5% | 184,7% |
| Urban land occupation | 110,6% | 109,2% | 110,0% | 101,0% | 100,1% | 100,2% |
| Water depletion | 723,6% | 696,3% | 721,0% | 561,9% | 564,9% | 566,5% |
| Freshwater eutrophication | 135,7% | 129,1% | 132,5% | 161,0% | 160,8% | 161,2% |
| Ozone depletion | 179,4% | 176,3% | 178,4% | 187,4% | 186,2% | 186,6% |
| Agricultural land occupation | 70,5% | 69,9% | 70,2% | 62,6% | 62,0% | 62,1% |

Table 14 shows the final midpoint comparison between the dynamic hourly grid resolution and the static reference system. The static system is calculated using the reference process for electricity production, and as such does not vary throughout the year and any kWh produced during the year is assumed to be equally impactful. Since the impact scores pr. kWh do not change, changing the building data resolution becomes redundant, and is instead calculated using the yearly total.

The differences in the impact score results are substantial, and are higher for all but one category; agricultural land occupation. For the categories where the results are higher, the difference ranges between 26,8% and all the way up to 687,1%. The extreme categories are ionizing radiation, freshwater ecotoxicity, marine ecotoxicity, and water depletion, these are the categories where the differences exceed 200%. The largest contributors to these categories are; waste, biogas, biogas, and electricity transformation, respectively.

The substantial differences are caused by a combination of the simplification of the data – and by extension the dynamic modelling practices, the inclusion of transformation processes – that for the dynamic grids are calculated from the Gross and Net production, and lastly the temporal differences of the grids – where the dynamic grids are from 2017 while the reference grid is from 2012.

Table 15: Differences in total endpoint scores pr. m2, between the various grid resolutions and reference grid, hourly and daily, hourly and monthly, hourly and reference.

| Hourly-Daily | W-I | W-II | W-III | E-I | E-II | E-III |
| --- | --- | --- | --- | --- | --- | --- |
| Ecosystems | 95,4% | 93,6% | 95,9% | 92,3% | 97,2% | 97,1% |
| Human Health | 96,2% | 94,2% | 96,9% | 91,6% | 96,3% | 96,4% |
| Resources | 98,2% | 96,1% | 98,9% | 92,9% | 97,6% | 97,8% |
| Hourly-Monthly | W-I | W-II | W-III | E-I | E-II | E-III |
| Ecosystems | 94,2% | 92,5% | 94,7% | 96,0% | 96,5% | 96,4% |
| Human Health | 98,9% | 95,7% | 98,5% | 97,7% | 98,0% | 98,0% |
| Resources | 100,8% | 97,6% | 100,3% | 99,1% | 99,3% | 99,5% |
| Hourly-Reference | W-I | W-II | W-III | E-I | E-II | E-III |
| Ecosystems | 105,5% | 103,8% | 105,2% | 97,8% | 97,0% | 97,1% |
| Human Health | 172,3% | 167,3% | 170,8% | 173,5% | 172,5% | 172,8% |
| Resources | 179,3% | 174,1% | 177,7% | 187,5% | 186,5% | 186,8% |

The same comparison was performed on the endpoint scores, they show a different trend with the hourly resolution having higher impact scores than the hourly and monthly resolutions, while the reference grid is once again producing results that are significantly higher.

The endpoint scores are summed up within the three categories as presented above, for easier presentation. While the differences as substantial, for the three major categories there are no results that differ over 100% as with the midpoints. This is due to the scores being summed up, when looking at the individual categories these extreme differences still occur, which means the results for the categories with the largest differences are minor compared to the other results within the sub-categories such as ecosystems for instance.

Table 16: Comparison between hourly resolution midpoint scores pr. m2, showing the differences in the age groups

| Hourly resolution midpoint scores | unit | W-I | W-II | W-III | E-I | E-II | E-III |
| --- | --- | --- | --- | --- | --- | --- | --- |
| Fossil depletion | kg oil eq /m2 | 2,64E+00 | 97,8% | 69,9% | 75,6% | 64,1% | 57,0% |
| Marine eutrophication | kg N eq /m2 | 2,49E-03 | 98,3% | 70,1% | 72,0% | 61,0% | 54,2% |
| Terrestrial acidification | kg SO2 eq /m2 | 4,00E-02 | 96,6% | 69,5% | 81,9% | 69,6% | 62,0% |
| Ionizing radiation | kg U235 eq /m2 | 1,04E+00 | 99,8% | 70,5% | 63,2% | 53,2% | 47,3% |
| Freshwater ecotoxicity | kg 1,4-DB eq /m2 | 1,71E-01 | 98,4% | 69,5% | 59,8% | 50,4% | 44,7% |
| Photochemical oxidant formation | kg NMVOC /m2 | 2,71E-02 | 96,7% | 69,5% | 75,8% | 64,4% | 57,3% |
| Terrestrial ecotoxicity | kg 1,4-DB eq /m2 | 1,45E-03 | 95,6% | 68,2% | 71,1% | 60,2% | 53,3% |
| Marine ecotoxicity | kg 1,4-DB eq /m2 | 1,61E-01 | 98,3% | 69,5% | 60,6% | 51,1% | 45,3% |
| Climate Change | kg CO2 eq /m2 | 1,01E+01 | 97,8% | 70,0% | 79,0% | 67,1% | 59,7% |
| Particulate matter formation | kg PM10 eq /m2 | 1,31E-02 | 96,8% | 69,4% | 80,6% | 68,4% | 60,9% |
| Human toxicity | kg 1,4-DB eq /m2 | 4,45E+00 | 99,0% | 70,3% | 69,6% | 58,9% | 52,3% |
| Metal depletion | kg Fe eq /m2 | 3,78E-01 | 97,7% | 69,2% | 60,7% | 51,2% | 45,5% |
| Natural land transformation | m2 /m2 | 1,43E-03 | 97,1% | 69,5% | 77,8% | 65,9% | 58,6% |
| Urban land occupation | m2*a /m2 | 2,15E-01 | 96,3% | 69,5% | 85,9% | 73,0% | 65,0% |
| Water depletion | m3 /m2 | 2,90E+01 | 98,9% | 69,3% | 100,9% | 84,7% | 75,3% |
| Freshwater eutrophication | kg P eq /m2 | 5,89E-03 | 100,0% | 70,8% | 66,1% | 55,8% | 49,6% |
| Ozone depletion | kg CFC-11 eq /m2 | 1,35E-06 | 96,8% | 69,5% | 75,0% | 63,7% | 56,7% |
| Agricultural land occupation | m2*a /m2 | 1,63E+01 | 96,0% | 69,4% | 88,3% | 75,2% | 66,9% |
| Average | - | - | 97,7% | 69,6% | 74,7% | 63,2% | 56,2% |
|  |  |  |  |  |  |  |  |

The scores presented in table 16 above illustrate the findings from the earlier sections, that the western buildings consume more electricity/m2 and as such have higher impact score results, and that the younger buildings generally use more electricity than the older, and as such have higher impact scores.

The table shows the total midpoint scores for group W-I, and the differences between age group W-I and the remaining groups as a percentage.

# Appendix A – midpoint scores

## Hourly grid resolution

Table 17: Midpoint impact score results from analysis of hourly grid resolution

| Hourly grid resolution | W-II | W-II | W-III | E-I | E-II | EI-III | unit |
| --- | --- | --- | --- | --- | --- | --- | --- |
| Fossil depletion | 2,64E+00 | 2,59E+00 | 1,85E+00 | 2,00E+00 | 1,69E+00 | 1,56E+00 | kg oil eq /m2 |
| Marine eutrophication | 2,49E-03 | 2,45E-03 | 1,75E-03 | 1,80E-03 | 1,52E-03 | 1,40E-03 | kg N eq /m2 |
| Terrestrial acidification | 4,00E-02 | 3,86E-02 | 2,78E-02 | 3,28E-02 | 2,78E-02 | 2,57E-02 | kg SO2 eq /m2 |
| Ionizing radiation | 1,04E+00 | 1,04E+00 | 7,34E-01 | 6,58E-01 | 5,53E-01 | 5,02E-01 | kg U235 eq /m2 |
| Freshwater ecotoxicity | 1,71E-01 | 1,68E-01 | 1,19E-01 | 1,02E-01 | 8,64E-02 | 7,86E-02 | kg 1,4-DB eq /m2 |
| Photochemical oxidant formation | 2,71E-02 | 2,63E-02 | 1,89E-02 | 2,06E-02 | 1,75E-02 | 1,61E-02 | kg NMVOC /m2 |
| Terrestrial ecotoxicity | 1,45E-03 | 1,39E-03 | 9,91E-04 | 1,03E-03 | 8,75E-04 | 8,01E-04 | kg 1,4-DB eq /m2 |
| Marine ecotoxicity | 1,61E-01 | 1,58E-01 | 1,12E-01 | 9,75E-02 | 8,22E-02 | 7,48E-02 | kg 1,4-DB eq /m2 |
| Climate Change | 1,01E+01 | 9,84E+00 | 7,04E+00 | 7,95E+00 | 6,75E+00 | 6,21E+00 | kg CO2 eq /m2 |
| Particulate matter formation | 1,31E-02 | 1,26E-02 | 9,06E-03 | 1,05E-02 | 8,93E-03 | 8,22E-03 | kg PM10 eq /m2 |
| Human toxicity | 4,45E+00 | 4,41E+00 | 3,13E+00 | 3,10E+00 | 2,62E+00 | 2,40E+00 | kg 1,4-DB eq /m2 |
| Metal depletion | 3,78E-01 | 3,69E-01 | 2,61E-01 | 2,29E-01 | 1,93E-01 | 1,76E-01 | kg Fe eq /m2 |
| Natural land transformation | 1,43E-03 | 1,39E-03 | 9,97E-04 | 1,12E-03 | 9,45E-04 | 8,66E-04 | m2 /m2 |
| Urban land occupation | 2,15E-01 | 2,07E-01 | 1,49E-01 | 1,85E-01 | 1,57E-01 | 1,45E-01 | m2*a /m2 |
| Water depletion | 2,90E+01 | 2,87E+01 | 2,01E+01 | 2,93E+01 | 2,46E+01 | 2,21E+01 | m3 / m2 |
| Freshwater eutrophication | 5,89E-03 | 5,89E-03 | 4,17E-03 | 3,89E-03 | 3,29E-03 | 3,01E-03 | kg P eq /m2 |
| Ozone depletion | 1,35E-06 | 1,31E-06 | 9,39E-07 | 1,01E-06 | 8,61E-07 | 7,93E-07 | kg CFC-11 eq /m2 |
| Agricultural land occupation | 1,63E+01 | 1,57E+01 | 1,13E+01 | 1,44E+01 | 1,23E+01 | 1,14E+01 | m2*a /m2 |

## Daily grid resolution

Table 18: Midpoint impact score results from analysis of daily grid resolution

| Daily grid resolution | WI | WII | WIII | EI | EII | EIII | unit |
| --- | --- | --- | --- | --- | --- | --- | --- |
| Fossil depletion | 2,67E+00 | 2,56E+00 | 1,87E+00 | 2,01E+00 | 1,70E+00 | 1,52E+00 | kg oil eq /m2 |
| Marine eutrophication | 2,51E-03 | 2,42E-03 | 1,77E-03 | 1,80E-03 | 1,53E-03 | 1,36E-03 | kg N eq /m2 |
| Terrestrial acidification | 4,01E-02 | 3,81E-02 | 2,80E-02 | 3,27E-02 | 2,79E-02 | 2,48E-02 | kg SO2 eq /m2 |
| Ionizing radiation | 1,06E+00 | 1,03E+00 | 7,48E-01 | 6,70E-01 | 5,64E-01 | 5,04E-01 | kg U235 eq /m2 |
| Freshwater ecotoxicity | 1,70E-01 | 1,65E-01 | 1,19E-01 | 1,02E-01 | 8,63E-02 | 7,73E-02 | kg 1,4-DB eq /m2 |
| Photochemical oxidant formation | 2,73E-02 | 2,59E-02 | 1,90E-02 | 2,21E-02 | 1,88E-02 | 1,67E-02 | kg NMVOC /m2 |
| Terrestrial ecotoxicity | 1,39E-03 | 1,33E-03 | 9,65E-04 | 1,01E-03 | 8,54E-04 | 7,60E-04 | kg 1,4-DB eq /m2 |
| Marine ecotoxicity | 1,60E-01 | 1,55E-01 | 1,12E-01 | 9,73E-02 | 8,21E-02 | 7,35E-02 | kg 1,4-DB eq /m2 |
| Climate Change | 1,02E+01 | 9,76E+00 | 7,15E+00 | 7,96E+00 | 6,77E+00 | 6,03E+00 | kg CO2 eq /m2 |
| Particulate matter formation | 1,23E-02 | 1,17E-02 | 8,58E-03 | 1,01E-02 | 8,59E-03 | 7,65E-03 | kg PM10 eq /m2 |
| Human toxicity | 4,49E+00 | 4,37E+00 | 3,19E+00 | 3,10E+00 | 2,62E+00 | 2,34E+00 | kg 1,4-DB eq /m2 |
| Metal depletion | 3,77E-01 | 3,62E-01 | 2,62E-01 | 2,34E-01 | 1,96E-01 | 1,76E-01 | kg Fe eq /m2 |
| Natural land transformation | 1,44E-03 | 1,37E-03 | 1,00E-03 | 1,13E-03 | 9,55E-04 | 8,50E-04 | m2 /m2 |
| Urban land occupation | 2,17E-01 | 2,05E-01 | 1,51E-01 | 1,84E-01 | 1,58E-01 | 1,40E-01 | m2*a /m2 |
| Water depletion | 3,05E+01 | 2,94E+01 | 2,12E+01 | 2,94E+01 | 2,47E+01 | 2,20E+01 | m3 / m2 |
| Freshwater eutrophication | 5,99E-03 | 5,87E-03 | 4,28E-03 | 3,91E-03 | 3,31E-03 | 2,96E-03 | kg P eq /m2 |
| Ozone depletion | 1,35E-06 | 1,28E-06 | 9,38E-07 | 1,02E-06 | 8,65E-07 | 7,70E-07 | kg CFC-11 eq /m2 |
| Agricultural land occupation | 1,64E+01 | 1,55E+01 | 1,14E+01 | 1,44E+01 | 1,23E+01 | 1,09E+01 | m2*a /m2 |

## Monthly grid resolution

Table 19: Midpoint impact score results from analysis of monthly grid resolution

| Monthly grid resolution | WI | WII | WIII | EI | EII | EIII | unit |
| --- | --- | --- | --- | --- | --- | --- | --- |
| Fossil depletion | 2,76E+00 | 2,62E+00 | 1,91E+00 | 2,04E+00 | 1,73E+00 | 1,55E+00 | kg oil eq /m2 |
| Marine eutrophication | 2,66E-03 | 2,52E-03 | 1,84E-03 | 1,86E-03 | 1,58E-03 | 1,40E-03 | kg N eq /m2 |
| Terrestrial acidification | 3,97E-02 | 3,77E-02 | 2,76E-02 | 3,25E-02 | 2,77E-02 | 2,47E-02 | kg SO2 eq /m2 |
| Ionizing radiation | 1,17E+00 | 1,11E+00 | 8,08E-01 | 7,17E-01 | 6,01E-01 | 5,37E-01 | kg U235 eq /m2 |
| Freshwater ecotoxicity | 1,84E-01 | 1,74E-01 | 1,27E-01 | 1,07E-01 | 9,03E-02 | 8,06E-02 | kg 1,4-DB eq /m2 |
| Photochemical oxidant formation | 2,70E-02 | 2,57E-02 | 1,88E-02 | 2,20E-02 | 1,87E-02 | 1,67E-02 | kg NMVOC /m2 |
| Terrestrial ecotoxicity | 1,39E-03 | 1,33E-03 | 9,66E-04 | 1,00E-03 | 8,50E-04 | 7,57E-04 | kg 1,4-DB eq /m2 |
| Marine ecotoxicity | 1,72E-01 | 1,64E-01 | 1,19E-01 | 1,02E-01 | 8,58E-02 | 7,66E-02 | kg 1,4-DB eq /m2 |
| Climate Change | 1,05E+01 | 9,94E+00 | 7,27E+00 | 8,08E+00 | 6,88E+00 | 6,13E+00 | kg CO2 eq /m2 |
| Particulate matter formation | 1,22E-02 | 1,16E-02 | 8,49E-03 | 1,01E-02 | 8,57E-03 | 7,63E-03 | kg PM10 eq /m2 |
| Human toxicity | 4,89E+00 | 4,63E+00 | 3,38E+00 | 3,25E+00 | 2,75E+00 | 2,46E+00 | kg 1,4-DB eq /m2 |
| Metal depletion | 3,92E-01 | 3,73E-01 | 2,71E-01 | 2,37E-01 | 1,99E-01 | 1,78E-01 | kg Fe eq /m2 |
| Natural land transformation | 1,45E-03 | 1,38E-03 | 1,00E-03 | 1,13E-03 | 9,57E-04 | 8,52E-04 | m2 /m2 |
| Urban land occupation | 2,11E-01 | 2,00E-01 | 1,47E-01 | 1,82E-01 | 1,56E-01 | 1,38E-01 | m2*a /m2 |
| Water depletion | 3,13E+01 | 2,99E+01 | 2,15E+01 | 3,01E+01 | 2,53E+01 | 2,25E+01 | m3 / m2 |
| Freshwater eutrophication | 6,70E-03 | 6,35E-03 | 4,64E-03 | 4,21E-03 | 3,55E-03 | 3,17E-03 | kg P eq /m2 |
| Ozone depletion | 1,35E-06 | 1,28E-06 | 9,39E-07 | 1,02E-06 | 8,65E-07 | 7,70E-07 | kg CFC-11 eq /m2 |
| Agricultural land occupation | 1,58E+01 | 1,50E+01 | 1,10E+01 | 1,41E+01 | 1,21E+01 | 1,07E+01 | m2*a /m2 |

## Reference grid

Table 20: Midpoint impact score results from analysis of reference grid

| Reference grid | WI | WII | WIII | EI | EII | EIII | unit |
| --- | --- | --- | --- | --- | --- | --- | --- |
| Fossil depletion | 4,80E+00 | 4,57E+00 | 3,32E+00 | 3,76E+00 | 3,17E+00 | 2,83E+00 | kg oil eq /m2 |
| Marine eutrophication | 3,49E-03 | 3,32E-03 | 2,41E-03 | 2,73E-03 | 2,31E-03 | 2,06E-03 | kg N eq /m2 |
| Terrestrial acidification | 6,05E-02 | 5,76E-02 | 4,18E-02 | 4,74E-02 | 4,00E-02 | 3,56E-02 | kg SO2 eq /m2 |
| Ionizing radiation | 5,82E+00 | 5,54E+00 | 4,02E+00 | 4,56E+00 | 3,85E+00 | 3,43E+00 | kg U235 eq /m2 |
| Freshwater ecotoxicity | 1,03E+00 | 9,78E-01 | 7,10E-01 | 8,06E-01 | 6,80E-01 | 6,06E-01 | kg 1,4-DB eq /m2 |
| Photochemical oxidant formation | 3,50E-02 | 3,33E-02 | 2,42E-02 | 2,74E-02 | 2,31E-02 | 2,06E-02 | kg NMVOC /m2 |
| Terrestrial ecotoxicity | 2,77E-03 | 2,63E-03 | 1,91E-03 | 2,17E-03 | 1,83E-03 | 1,63E-03 | kg 1,4-DB eq /m2 |
| Marine ecotoxicity | 9,07E-01 | 8,63E-01 | 6,27E-01 | 7,11E-01 | 6,00E-01 | 5,35E-01 | kg 1,4-DB eq /m2 |
| Climate Change | 1,89E+01 | 1,80E+01 | 1,31E+01 | 1,48E+01 | 1,25E+01 | 1,11E+01 | kg CO2 eq /m2 |
| Particulate matter formation | 1,90E-02 | 1,81E-02 | 1,31E-02 | 1,49E-02 | 1,26E-02 | 1,12E-02 | kg PM10 eq /m2 |
| Human toxicity | 7,20E+00 | 6,85E+00 | 4,97E+00 | 5,64E+00 | 4,76E+00 | 4,24E+00 | kg 1,4-DB eq /m2 |
| Metal depletion | 8,57E-01 | 8,16E-01 | 5,92E-01 | 6,72E-01 | 5,67E-01 | 5,05E-01 | kg Fe eq /m2 |
| Natural land transformation | 2,64E-03 | 2,51E-03 | 1,82E-03 | 2,07E-03 | 1,74E-03 | 1,55E-03 | m2 /m2 |
| Urban land occupation | 2,38E-01 | 2,26E-01 | 1,64E-01 | 1,87E-01 | 1,57E-01 | 1,40E-01 | m2*a /m2 |
| Water depletion | 2,10E+02 | 2,00E+02 | 1,45E+02 | 1,64E+02 | 1,39E+02 | 1,24E+02 | m3 / m2 |
| Freshwater eutrophication | 8,00E-03 | 7,61E-03 | 5,53E-03 | 6,27E-03 | 5,29E-03 | 4,71E-03 | kg P eq /m2 |
| Ozone depletion | 2,42E-06 | 2,31E-06 | 1,67E-06 | 1,90E-06 | 1,60E-06 | 1,43E-06 | kg CFC-11 eq /m2 |
| Agricultural land occupation | 1,15E+01 | 1,10E+01 | 7,96E+00 | 9,03E+00 | 7,62E+00 | 6,79E+00 | m2*a /m2 |

# Appendix B – Endpoint scores

## Hourly grid resolution

Table 21: Endpoint impact score results from analysis of hourly grid resolution

| Hourly grid resolution | W-I | W-II | W-III | E-I | E-II | E-III | Unit |
| --- | --- | --- | --- | --- | --- | --- | --- |
| Ecosystems | 3,03E-07 | 2,93E-07 | 2,10E-07 | 2,56E-07 | 2,18E-07 | 1,94E-07 | Species/year/m2 |
| Human Health | 2,13E-05 | 2,08E-05 | 1,48E-05 | 1,65E-05 | 1,40E-05 | 1,25E-05 | DALY/m2 |
| Resources | 4,77E-01 | 4,68E-01 | 3,33E-01 | 3,58E-01 | 3,03E-01 | 2,70E-01 | $/m2 |

## Daily grid resolution

Table 22: Endpoint impact score results from analysis of daily grid resolution

| Daily grid resolution | W-I | W-II | W-III | E-I | E-II | E-III |  |
| --- | --- | --- | --- | --- | --- | --- | --- |
| Ecosystems | 2,89E-07 | 2,74E-07 | 2,01E-07 | 2,36E-07 | 2,12E-07 | 1,88E-07 | Species/year/m2 |
| Human Health | 2,04E-05 | 1,96E-05 | 1,44E-05 | 1,52E-05 | 1,35E-05 | 1,20E-05 | DALY/m2 |
| Resources | 4,69E-01 | 4,49E-01 | 3,29E-01 | 3,33E-01 | 2,96E-01 | 2,64E-01 | $/m2 |

## Monthly grid resolution

Table 23: Endpoint impact score results from analysis of monthly grid resolution

| Monthly grid resolution | W-I | W-II | W-III | E-I | E-II | E-III |  |
| --- | --- | --- | --- | --- | --- | --- | --- |
| Ecosystems | 2,86E-07 | 2,71E-07 | 1,99E-07 | 2,46E-07 | 2,10E-07 | 1,87E-07 | Species/year/m2 |
| Human Health | 2,10E-05 | 1,99E-05 | 1,46E-05 | 1,62E-05 | 1,37E-05 | 1,22E-05 | DALY/m2 |
| Resources | 4,81E-01 | 4,56E-01 | 3,34E-01 | 3,55E-01 | 3,01E-01 | 2,69E-01 | $/m2 |

## Reference grid

Table 24: Endpoint score results from analysis of reference grid

| Reference grid | W-I | W-II | W-III | E-I | E-II | E-III |  |
| --- | --- | --- | --- | --- | --- | --- | --- |
| Ecosystems | 3,20E-07 | 3,04E-07 | 2,21E-07 | 2,50E-07 | 2,11E-07 | 1,88E-07 | Species/year/m2 |
| Human Health | 3,66E-05 | 3,48E-05 | 2,53E-05 | 2,87E-05 | 2,42E-05 | 2,16E-05 | DALY/m2 |
| Resources | 8,56E-01 | 8,14E-01 | 5,91E-01 | 6,71E-01 | 5,66E-01 | 5,04E-01 | $/m2 |

# Appendix C – Reference unit impacts & Impact potentials

Table 1: Reference source specific unit impacts potentials from openLCA, showing the impacts pr. kWh for every electricity production source

|  | **Source specific unit impacts** | | | | | | | | | | |  |
| --- | --- | --- | --- | --- | --- | --- | --- | --- | --- | --- | --- | --- |
| **Impact category** | **Oil** | **Coal** | **Waste** | **Solar** | **Wind Offshore** | **Wind Onshore** | **Natural Gas** | **Biomass** | **Bio gas** | **Exchange Continent** | **Exchange Nordics** | **Unit** |
| Fossil depletion | 3,20x10^-1^ | 2,21x10^-1^ | 2,27x10^-1^ | 2,60x10^-2^ | 3,83x10^-3^ | 3,38x10^-3^ | 1,88x10^-1^ | 1,45x10^-2^ | 3,38x10^-2^ | 1,69x10^-1^ | 7,30x10^-3^ | kg oil eq/kWh |
| Marine eutrophication | 2,10x10^-4^ | 1,10x10^-4^ | 2,50x10^-4^ | 4,58x10^-5^ | 6,47x10^-6^ | 4,30x10^-6^ | 1,28x10^-5^ | 8,67x10^-5^ | 2,40x10^-4^ | 2,10x10^-4^ | 3,99x10^-6^ | kg N eq/kWh |
| Terrestrial acidification | 1,23x10^-2^ | 2,37x10^-3^ | 4,42x10^-3^ | 6,20x10^-4^ | 8,13x10^-5^ | 6,51x10^-5^ | 2,20x10^-4^ | 1,60x10^-3^ | 1,38x10^-3^ | 9,60x10^-4^ | 8,13x10^-5^ | kg SO2 eq/kWh |
| Ionizing radiation | 6,38x10^-2^ | 3,62x10^-3^ | 6,64x10^-1^ | 1,13x10^-2^ | 8,90x10^-4^ | 8,50x10^-4^ | 1,55x10^-3^ | 3,24x10^-3^ | 1,10x10^-2^ | 1,29x10^-1^ | 1,04x10^-2^ | kg U235 eq/kWh |
| Freshwater ecotoxicity | 1,21x10^-3^ | 5,88x10^-3^ | 4,77x10^-3^ | 1,51x10^-2^ | 1,83x10^-3^ | 3,18x10^-3^ | 2,60x10^-4^ | 1,08x10^-3^ | 2,34x10^-2^ | 1,39x10^-2^ | 2,90x10^-4^ | kg 1,4-DB eq/kWh |
| Photochemical oxidant formation | 4,14x10^-3^ | 1,05x10^-3^ | 2,68x10^-3^ | 4,00x10^-4^ | 5,97x10^-5^ | 5,54x10^-5^ | 3,60x10^-4^ | 1,77x10^-3^ | 1,00x10^-3^ | 7,10x10^-4^ | 6,45x10^-5^ | kg NMVOC/kWh |
| Terrestrial ecotoxicity | 9,42x10^-5^ | 6,42x10^-6^ | 8,20x10^-4^ | 1,80x10^-4^ | 2,54x10^-6^ | 1,84x10^-6^ | 2,02x10^-6^ | 7,82x10^-5^ | 2,00x10^-4^ | 1,76x10^-5^ | 1,38x10^-6^ | kg 1,4-DB eq/kWh |
| Marine ecotoxicity | 1,86x10^-3^ | 5,64x10^-3^ | 5,10x10^-3^ | 1,38x10^-2^ | 1,66x10^-3^ | 2,79x10^-3^ | 5,10x10^-4^ | 1,25x10^-3^ | 2,04x10^-2^ | 1,32x10^-2^ | 2,70x10^-4^ | kg 1,4-DB eq/kWh |
| Climate Change | 9,68x10^-1^ | 1,04 | 6,31x10^-1^ | 1,00x10^-1^ | 1,58x10^-2^ | 1,26x10^-2^ | 4,44x10^-1^ | 5,56x10^-2^ | 2,20x10^-1^ | 6,48x10^-1^ | 2,78x10^-2^ | kg CO2 eq/kWh |
| Particulate matter formation | 2,97x10^-3^ | 6,50x10^-4^ | 1,50x10^-3^ | 3,20x10^-4^ | 5,24x10^-5^ | 4,53x10^-5^ | 1,10x10^-4^ | 5,40x10^-4^ | 4,90x10^-4^ | 3,60x10^-4^ | 5,21x10^-5^ | kg PM10 eq/kWh |
| Human toxicity | 6,58x10^-2^ | 2,34x10^-1^ | 1,59x10^-1^ | 1,52x10^-1^ | 1,84x10^-2^ | 1,53x10^-2^ | 7,06x10^-3^ | 1,11x10^-1^ | 1,59x10^-1^ | 4,99x10^-1^ | 7,30x10^-3^ | kg 1,4-DB eq/kWh |
| Metal depletion | 5,13x10^-3^ | 3,59x10^-3^ | 6,81x10^-2^ | 2,91x10^-2^ | 1,17x10^-2^ | 8,61x10^-3^ | 2,64x10^-3^ | 5,13x10^-3^ | 9,17x10^-3^ | 1,22x10^-2^ | 2,79x10^-3^ | kg Fe eq/kWh |
| Natural land transformation | 3,50x10^-4^ | 4,08x10^-5^ | 2,90x10^-4^ | 1,60x10^-5^ | 2,13x10^-6^ | 1,79x10^-6^ | 1,40x10^-4^ | 2,06x10^-5^ | 3,86x10^-5^ | 4,10x10^-5^ | 1,59x10^-5^ | m2/kWh |
| Urban land occupation | 1,57x10^-3^ | 8,42x10^-3^ | 5,12x10^-3^ | 1,14x10^-3^ | 1,90x10^-4^ | 1,05x10^-3^ | 3,00x10^-4^ | 2,14x10^-2^ | 2,77x10^-3^ | 2,71x10^-3^ | 2,70x10^-4^ | m2*a/kWh |
| Water depletion | 2,69x10^-1^ | 2,17x10^-1^ | 6,34x10^-1^ | 1,61 | 1,05x10^-1^ | 6,80x10^-2^ | 1,48x10^-1^ | 6,20x10^-2^ | 4,08x10^-1^ | 2,22 | 1,32 | m3/kWh |
| Freshwater eutrophication | 1,97x10^-5^ | 3,70x10^-4^ | 7,68x10^-5^ | 9,20x10^-5^ | 1,03x10^-5^ | 8,95x10^-6^ | 6,43x10^-6^ | 3,87x10^-5^ | 6,30x10^-4^ | 8,50x10^-4^ | 7,17x10^-6^ | kg P eq/kWh |
| Ozone depletion | 1,72x10^-7^ | 3,40x10^-9^ | 5,01x10^-7^ | 2,12x10^-8^ | 1,25x10^-9^ | 1,13x10^-9^ | 4,43x10^-8^ | 9,41x10^-8^ | 1,24x10^-8^ | 3,79x10^-8^ | 3,46x10^-9^ | kg CFC-11 eq/kWh |
| Agricultural land occupation | 2,43x10^-3^ | 1,31x10^-2^ | 3,77x10^-1^ | 7,83x10^-3^ | 6,10x10^-4^ | 5,00x10^-4^ | 8,30x10^-4^ | 2,31 | 2,32x10^-2^ | 6,04x10^-2^ | 9,68x10^-3^ | m2*a/kWh |

Table 2: Impact potentials pr. kWh by months relative to the yearly average, West - Hourly. The months with impacts significantly lower than the yearly average are denoted by a green color, while the higher scores are presented as orange/red.

|  | January | February | March | April | May | June | July | August | September | October | November | December |
| --- | --- | --- | --- | --- | --- | --- | --- | --- | --- | --- | --- | --- |
| Fossil depletion | 117,2% | 122,8% | 119,1% | 105,2% | 96,0% | 61,4% | 55,8% | 101,2% | 103,5% | 102,4% | 104,5% | 110,8% |
| Marine eutrophication | 108,4% | 119,5% | 114,9% | 104,8% | 95,0% | 64,6% | 55,0% | 109,7% | 106,2% | 107,9% | 101,7% | 112,2% |
| Terrestrial acidification | 137,1% | 133,6% | 130,0% | 108,7% | 99,3% | 52,5% | 53,4% | 81,6% | 96,5% | 90,8% | 108,6% | 107,8% |
| Ionizing radiation | 78,3% | 94,6% | 95,5% | 96,4% | 92,6% | 88,1% | 76,2% | 134,9% | 114,6% | 119,4% | 98,2% | 111,1% |
| Freshwater ecotoxicity | 86,7% | 105,9% | 101,6% | 106,2% | 97,8% | 90,6% | 75,4% | 118,3% | 101,1% | 115,0% | 94,3% | 107,1% |
| Photochemical oxidant formation | 135,2% | 132,3% | 128,8% | 108,4% | 99,1% | 53,9% | 54,4% | 83,1% | 96,9% | 91,7% | 108,3% | 107,9% |
| Terrestrial ecotoxicity | 117,8% | 114,4% | 120,4% | 108,7% | 107,7% | 76,0% | 79,4% | 90,9% | 92,9% | 90,8% | 104,3% | 96,7% |
| Marine ecotoxicity | 87,7% | 106,5% | 102,3% | 106,1% | 97,8% | 89,2% | 74,3% | 118,1% | 101,6% | 114,6% | 94,5% | 107,3% |
| Climate Change | 120,8% | 126,3% | 121,2% | 106,2% | 96,5% | 57,4% | 51,8% | 99,2% | 104,7% | 101,0% | 103,8% | 111,2% |
| Particulate matter formation | 129,7% | 127,7% | 125,0% | 107,5% | 99,8% | 59,9% | 60,1% | 86,4% | 97,8% | 93,0% | 106,7% | 106,5% |
| Human toxicity | 96,5% | 114,5% | 108,6% | 104,1% | 94,6% | 69,7% | 55,2% | 121,2% | 111,1% | 114,4% | 96,9% | 113,2% |
| Metal depletion | 91,4% | 103,2% | 99,8% | 105,0% | 99,1% | 103,1% | 93,4% | 104,5% | 92,2% | 109,1% | 97,5% | 101,8% |
| Natural land transformation | 116,5% | 111,2% | 114,8% | 101,2% | 99,2% | 75,1% | 79,0% | 95,8% | 100,6% | 95,1% | 107,9% | 103,6% |
| Urban land occupation | 147,7% | 142,0% | 135,8% | 111,2% | 99,5% | 45,2% | 47,0% | 72,3% | 94,0% | 86,8% | 109,7% | 108,7% |
| Water depletion | 70,5% | 64,8% | 78,6% | 86,6% | 108,1% | 116,9% | 126,5% | 135,9% | 132,1% | 99,3% | 90,0% | 90,7% |
| Freshwater eutrophication | 88,0% | 111,1% | 104,4% | 102,1% | 91,5% | 70,3% | 51,8% | 131,7% | 115,7% | 121,1% | 95,7% | 116,6% |
| Ozone depletion | 127,2% | 124,7% | 124,2% | 106,1% | 98,1% | 61,8% | 62,4% | 88,4% | 95,8% | 94,8% | 109,5% | 107,2% |
| Agricultural land occupation | 156,3% | 146,3% | 141,1% | 111,6% | 100,2% | 38,4% | 43,5% | 66,4% | 93,0% | 82,4% | 112,6% | 108,3% |
